# Health-related quality of life in adults with low-grade gliomas: a systematic review

**DOI:** 10.1007/s11136-022-03207-x

**Published:** 2022-08-06

**Authors:** Ben Rimmer, Iakov Bolnykh, Lizzie Dutton, Joanne Lewis, Richéal Burns, Pamela Gallagher, Sophie Williams, Vera Araújo-Soares, Fiona Menger, Linda Sharp

**Affiliations:** 1 Population Health Sciences Institute, Newcastle University, Newcastle University Centre for Cancer, Ridley Building 1, Newcastle upon Tyne, NE1 7RU Newcastle, England; 2 Faculty of Medical Sciences, Newcastle University, Newcastle, England; 3 Newcastle Upon Tyne Hospitals NHS Foundation Trust, Newcastle, England; 4 Department of Health and Nutritional Sciences, Sligo, IT Ireland; 5 School of Psychology, Dublin City University, Dublin, Ireland; 6 Faculty of Behavioural, Management and Social Sciences, Department of Health Technology and Services Research, University of Twente, Twente, The Netherlands; 7 School of Education, Communication and Language Sciences, Newcastle University, Newcastle, England

**Keywords:** Low-grade glioma, Health-related quality-of-life, Survivorship

## Abstract

**Purpose:**

Low-grade glioma (LGG) patients may face health-related quality-of-life (HRQoL) impairments, due to the tumour, treatment and associated side-effects and prospects of progression. We systematically identified quantitative studies assessing HRQoL in adult LGG patients, for: aspects of HRQoL impacted; comparisons with non-cancer controls (NCC) and other groups; temporal trends; and factors associated with HRQoL.

**Methods:**

MEDLINE, CINAHL, Embase, PubMed, and PsycINFO were systematically searched from inception to 14th September 2021. Following independent screening of titles and abstracts and full-texts, population and study characteristics, and HRQoL findings were abstracted from eligible papers, and quality appraised. Narrative synthesis was conducted.

**Results:**

Twenty-nine papers reporting 22 studies (cross-sectional, *n* = 13; longitudinal, *n* = 9) were identified. Papers were largely good quality, though many excluded patients with cognitive and communication impairments. Comparators included high-grade gliomas (HGG) (*n* = 7); NCCs (*n* = 6) and other patient groups (*n* = 3). Nineteen factors, primarily treatment (n = 8), were examined for association with HRQoL. There was substantial heterogeneity in HRQoL instruments used, factors and aspects of HRQoL assessed and measurement timepoints. HRQoL, primarily cognitive functioning and fatigue, in adult LGG patients is poor, and worse than in NCCs, though better than in HGG patients. Over time, HRQoL remained low, but stable. Epilepsy/seizure burden was most consistently associated with worse HRQoL.

**Conclusion:**

LGG patients experience wide-ranging HRQoL impairments. HRQoL in those with cognitive and communication impairments requires further investigation. These findings may help clinicians recognise current supportive care needs and inform types and timings of support needed, as well as inform future interventions.

**Supplementary Information:**

The online version contains supplementary material available at 10.1007/s11136-022-03207-x.

## Plain English Summary

Low-grade gliomas are brain tumours most commonly diagnosed in working-aged adults. Brain tumour patients can experience numerous symptoms, such as communication impairment and mobility issues, which can impact their quality of life. Patients with low-grade gliomas have a longer life expectancy than patients with other, high-grade brain tumours, though they are rarely cured. Therefore, it is important to understand how their quality of life is impacted in the extended periods living with a low-grade glioma. We looked at which aspects of health-related quality of life were impacted; how health-related quality of life compared with other patient populations; whether health-related quality of life changed over time; and whether any factors (e.g. age) influenced health-related quality of life. We found that low-grade glioma patients experience wide-ranging health-related quality of life impairments, particularly fatigue and cognitive impairment, that remains poor, but does not change much over time. Though better than in high-grade gliomas, health-related quality of life was worse than in people without cancer and was influenced by several factors, most frequently seizures. This means low-grade glioma patients may live for long periods with poor health-related quality of life. Our findings may help clinicians recognise what these patients’ supportive care needs are, and what support is needed.

## Introduction

Worldwide, in 2020, there were approximately 300,000 new brain and central nervous system tumours diagnosed [1]. Gliomas – which may be high- or low-grade—are the most common malignant tumour of the brain [2]. Low-grade gliomas (LGG) account for approximately 15% of all gliomas, with an incidence rate of around 1/100,000; they are mostly diagnosed in adults in their 30 s and 40 s [3]. Depending on the subtype, life expectancy of LGG patients is limited to about 5–15 years [3, 4]. However, LGGs are rarely cured, and typically recur or progress to a high-grade glioma (HGG) [5]. Thus, LGG patients may live for extended periods with a ‘terminal’ condition.

Health-related quality-of-life (HRQoL) is a multidimensional construct that comprises the ability to perform everyday activities, as well as patient satisfaction with levels of functioning and disease control [6]. Brain tumour patients can experience an array of symptoms, often occurring in clusters and deteriorating as the disease progresses [7]. These include general cancer-related symptoms (e.g. fatigue, pain), and tumour-specific symptoms (e.g. cognitive limitations, seizures, speech, language, and communication impairments, personality changes and mobility issues) [8–10]. These symptoms can contribute to changes in social roles, daily functioning, and loss of independence, which adversely impact physical and psychosocial HRQoL [10, 11].

Studies suggest there are numerous factors (e.g. age, tumour location, and time since diagnosis), that could influence brain tumour patients’ HRQoL [12]. Gaining a comprehensive understanding, from across the literature of how these factors are associated with HRQoL and how HRQoL changes over time, may help to ascertain in whom, what, and when, support is necessary and identify target areas for future interventions.

It is, however, difficult to distinguish the extent these problems are experienced by LGG patients. One issue is sample heterogeneity; studies often group patients with LGGs, HGGs, and other primary brain tumours [13–15]. This limits our understanding of the HRQoL impact of living long-term with a tumour that is still likely to progress. Further, much of the evidence comes from treatment trials. Trial populations are often highly selected and have a lower risk profile than ‘real-world’ patient populations [16]. Treatment modalities (e.g. surgery, radiotherapy, and chemotherapy) have been associated with HRQoL in LGG patients [17–19]. Thus, HRQoL impairments may be due to the tumour or its treatment. Consequently, there is a need to better understand the ‘real world’ impact of an LGG on HRQoL, outwith the trial context.

We, therefore, conducted a systematic review to examine how HRQoL is impacted in adults with an LGG, by establishing: (1) which aspects of HRQoL are impacted; (2) how HRQoL compares with other populations; (3) temporal trends in HRQoL; and (4) factors associated with HRQoL. Our secondary aims were to assess quality of, and identify gaps or limitations in, the available evidence.

## Methods

This systematic review was registered with the Prospective Register for Systematic Reviews (PROSPERO) (CRD42021231368) and conducted and reported in accordance with the Preferred Reporting Items for Systematic Review and Meta-Analysis (PRISMA) guidelines [20].

### Definition

For the purposes of this review, we defined HRQoL as “*the subjective perceptions of the positive and negative aspects of cancer patients’ symptoms, including physical, emotional, social, and cognitive functions and, importantly, disease symptoms and side effects of treatment*.”[21] Hereafter, ‘*global HRQoL*’ indicates total scores, while ‘*specific (aspects of) HRQoL*’ indicates functioning and symptoms.

### Eligibility criteria

A paper was eligible if: (1) it was a primary, peer-reviewed research article, available in English; (2) participants were adults (≥ 18-years old), diagnosed with an LGG; (3) data were from an observational study conducted in a ‘*real-world*’ setting (i.e. in routine practice, outwith the clinical trial context); (4) an instrument was used to quantitatively assess HRQoL, with evidence of content validity or other psychometric properties. Papers which focused on a single issue (e.g. psychological wellbeing) were eligible if the issue was framed, in the paper, as an aspect of HRQoL.

A paper was excluded if: (1) the sample was heterogenous (e.g. included HGGs) and LGGs were not a distinct group; (2) the HRQoL findings were not reported; (3) participants were adult survivors of childhood diagnoses (< 18-years); or (4) data were from a trial directly investigating specific treatments (e.g. impact of radiotherapy).

### Search strategy

On 10th December 2020, we searched five electronic databases from inception: MEDLINE, Embase, PsycINFO, CINAHL, and PubMed. The search strategy concerned two key concepts: LGG and HRQoL. Assisted by a Senior Library Assistant, a combination of Medical Subject Headings and keywords were formulated, informed by the literature (Supplementary Table S1). LGG was searched using general terms and specific tumours, in line with the 2016 WHO classification of tumours of the central nervous system [22]. The 2021 WHO classification update [23] succeeded initial database searches, though our search strategy still encompassed LGGs, as they are now classified. HRQoL was searched using general terms and terms for HRQoL instruments that were previously reported to have been used in brain tumour patients [24] (although studies did not have to have used these instruments to be eligible). The search strategy was adapted accordingly for each database (Supplementary Table S2).

Reference lists and forward citations of eligible papers and relevant reviews were hand-searched to identify additional papers not retrieved through the database searches. The search was updated on 14th September 2021.

### Paper selection

Once duplicates were removed, B.R and I.B independently screened titles and abstracts, followed by full texts of papers considered potentially eligible by either reviewer. The process was blinded until both reviewers completed each stage of screening. Discrepancies at paper selection were resolved through discussion with co-authors (L.D and L.S).

### Data extraction and quality appraisal

Data extraction was conducted and cross-checked (shared between B.R and I.B), using a structured form. The following data were extracted: *general:* name of first author, year published, country; *study population:* eligible population, sample size, participant characteristics, namely, age, sex, ethnicity, socio-economic status (SES), Karnofsky performance status (KPS), tumour type and location, genetic markers, treatment, time since diagnosis/treatment; *study design:* design, comparator/control populations, HRQoL measurement timepoints, HRQoL instrument(s) used and specific aspects of HRQoL assessed, clinical and epidemiological factors examined for association with HRQoL; *findings:* global HRQoL, specific HRQoL, HRQoL in comparators/controls, HRQoL over time (e.g. mean scores), and factors associated with HRQoL (e.g. correlation coefficients).

If more than one paper reported the same sample, then characteristics and findings were pooled as one study. Corresponding authors were contacted to request relevant missing information. No reply within three weeks meant data extraction decisions were informed by the available published material. Discrepancies at data extraction were resolved through discussion between co-authors (B.R and I.B).

Included papers were quality appraised and cross-checked (shared between B.R and I.B), using the 12-item critical appraisal checklist, established by Dunne et al. [25] in a previous systematic review on quality-of-life in cancer survivors. Items included ‘*main features of population/design described*’ and ‘*measures relevant, validated, and described adequately*’. Each item was scored 0 (no), 1 (partial) or 2 (yes). Potential scores ranged from 0–24, with 0–8 indicating ‘*low quality*’, 9–16 ‘*acceptable quality*’, and 17–24 ‘*good quality*’.

### Data synthesis and analysis

Eligible studies were included in a narrative synthesis [26]. This was structured around the study population, design, quality appraisal, and HRQoL assessment, namely: global and specific HRQoL, population comparisons, temporal trends, and associated factors. Aspects of HRQoL which are included in the relevant instrument(s), but which were not reported by authors, were abstracted as ‘not reported’.

To interpret HRQoL, we used previously reported reference values; these were available for EORTC QLQ-C30 [27], EQ-5D [28], and FACT-G [29]. Otherwise, judgements were based on interpretations of the original authors; here, to ensure consistency, a value interpreted as ‘*poor*’ in one study, was considered ‘*poor*’ across all other studies which used the same instrument (there were no instances of different interpretations for values by authors of the papers). To synthesise the interpreted values for specific aspects of HRQoL, studies were grouped when different studies/instruments reported a dimension with the same (e.g. fatigue) or similar label (e.g. emotional wellbeing/functioning). In the synthesis, papers were “weighted” equally irrespective of the quality appraisal results.

## Results

### Search results

**Fig. 1:**
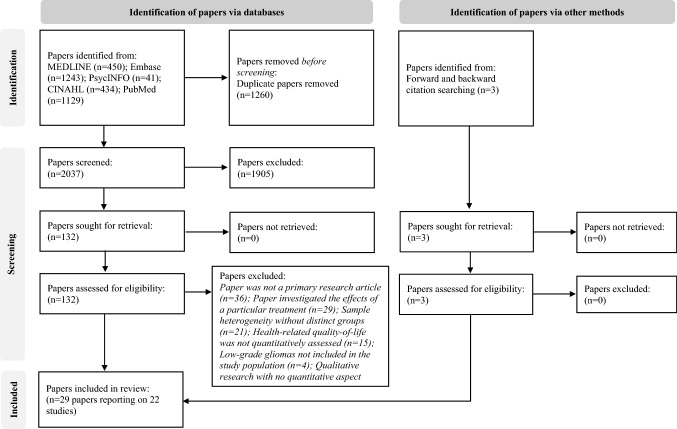
PRISMA flow diagram of paper selection for quantitative studies that assessed health-related quality-of-life in low-grade glioma patients

Database searches identified 3295 papers, with 2037 remaining following deduplication. Full texts of 132 papers were assessed for eligibility, with 26 papers deemed eligible. Hand searches identified three additional papers. Twenty-nine papers reporting on 22 studies were included [12, 30–57] (Fig. 1).

### Study population

Studies were conducted across 13 countries: three each in the Netherlands [12, 30–32, 44, 48] and USA [33, 36, 37, 39], two each in China [42, 54–56], Italy [35, 45], India [34, 46], Japan [47, 53], and Norway [38, 41], and one each in Australia [52], Finland [50, 51], Germany [49], South Korea [43], Sweden [40], and Turkey [57] (Table 1; Supplementary Table S3). Sample size ranged from 15 to 260. Mean age was typically late 30 s and 40 s. Sex ranged from 24 to 73% female. Only Affronti et al. [33] reported ethnicity, for a predominantly white (93%) sample. Eleven studies reported SES [12, 35, 36, 39, 42, 44, 45, 48, 49, 54, 57] assessed through education, employment, or insurance status. Nine studies reported KPS [12, 33, 38, 41, 47–49, 54, 57]; scores ranged 60–100, but were mostly ≥ 80.

**Table 1:** Population characteristics of included quantitative studies that assessed health-related quality of life in low-grade glioma patients

Study (Country)	Sample size	Mean age (SD)^g^	% Female	Tumour type	Tumour location	Treatment	Time since diagnosis/ treatment
Aaronson (2011); Boele (2014; 2015); Ediebah (2017) (Netherlands)[12, 30–32]	T1: 195, T2: 65^a^	40.8 (11.6)	38.5%	Grade I (10.8%), Grade II (89.2%); Astrocytoma (71.3%), Oligodendroglioma (22%), Oligoastrocytoma (6.7%)	*Location:* frontal (24.1%), temporal (16.9%), parietal (9.7%), occipital (2.6%), mixed (45.6%), other (1%); *Laterality:* left (43.6%), Right (44.6%), Bilateral (4.6%)	Biopsy (43.1%), Resection (56.9%); Radiotherapy: yes (53.3%), no (46.7%)	*Since diagnosis:* mean 5.6 (SD 3.7) years
Affronti (2018) (USA) [33]	15	40 (11)	73%	Diffuse astrocytoma (53%), Infiltrating glioma (7%), Well-differentiated oligodendroglioma (40%)	Bilateral multifocal (7%), Left frontal (20%), Right frontal (53%), Right parietal (20%)	Biopsy (33%), Gross total resection (67%); Postsurgical temozolomide (40%)	*Since diagnosis:* 2 months at initial assessment
Budrukkar (2009) (India) [34]	71	NR^h^	NR^h^	NR	NR	NR	NR
Campanella (2017) (Italy) [35]	50	40 (10.9)	54%	NR	*Location:* frontal (60%), temporal (30%), parietal (10%); *Laterality:* right (50%), left (50%)	Surgery (100%), Radiotherapy (10%), Chemotherapy (10%), Both (4%)	*Since treatment:* mean 40.3 months, range 12–181 months
Correa (2007; 2008) (USA) [36, 37]	T1: 40, T2 and T3: 25^b^	41.5 (9.4)	37.5%	Oligodendroglioma (47.5%), Astrocytoma (22.5%), Oligoastrocytoma (25%), Not available (5%)	*Location:* frontal (37.5%), fronto-parietal (30%), temporal-parietal-occipital (27.5%), cortical/subcortical (5%); *Laterality:* right (57.5%), left (42.5%)	Resection (90%), Radiotherapy (30%), Chemotherapy (10%), No adjuvant therapy (60%)	*Since diagnosis:* median 71 months (27–142) (treated), 22 months (3–82) (untreated); *Since treatment:* median 38 months (6–118)
Drewes (2018) (Norway) [38]	40	46.7 (16.2)	32.5%	NR	*Laterality:* right (45%), left (40%), bilateral/midline (15%)	Preoperative corticosteroids (10%), Gross total resection (45%), Subtotal resection (42.5%), Biopsy only (12.5%)	*Since treatment:* 1–3 days before treatment at initial assessment
Gabel (2019) (USA) [39]	21	42.7 (13.6)	23.8%	NR	*Location:* frontal (23.8%), parietal (9.5%), temporal (19%), occipital (4.8%), insular (19%), other (23.8%); *Laterality:* left (42.9%), right (47.6%), midline (9.5%)	NR	NR
Gustafsson (2006) (Sweden) [40]	39	47 (14)	31%	Astrocytoma (*n* = 23), Oligodendroglioma (*n* = 8), Oligoastrocytoma (*n* = 4), Ependymoma (*n* = 3), Other (*n* = 1)	NR	Surgical resection (*n* = 29), Radiotherapy (*n* = 23), Chemotherapy (*n* = 8)	*Since diagnosis:* mean 16 years (< 1 to 47)
Jakola (2012) (Norway) [41]	55	41 (13)	45%	Astrocytoma (53%), Oligodendroglioma (29%), Oligoastrocytoma (18%)	NR	Biopsy (18%), Resection (82%), < 6 months post-op radiotherapy (26%), Radiotherapy (46%), < 6 months post-op chemotherapy (16%), Chemotherapy (33%), Later/repeat resection (22%)	*Since treatment:* mean 7 years
Jiang (2019) (China) [42]	219	41.5 (10.9)	38%	Astrocytoma (*n* = 103), Oligodendroglioma (*n* = 56), Oligoastrocytoma (*n* = 60)	*Location:* frontal (64%), non-frontal (36%); *Laterality:* right (38%), left (54%), bilateral (8%)	Resection: complete (83%), incomplete or biopsy (17%); Adjuvant therapy: yes (30%), no (70%)	*Since diagnosis:* > 3 months; *Since treatment:* 3 months
Kim (2020) (South Korea) [43]	45	NR^h^	NR^h^	Grade I (*n* = 13), II (*n* = 32)	NR^h^	NR^h^	*Since diagnosis:* > 3 months
Klein (2003) (Netherlands)[44]^c^	156 (21, 21, 33, 24, 24, 33)	35.8 (10.1), 37.7 (11.5), 43.4 (11.7), 43.5 (12.2), 45.7 (13.2), 41 (8.5)	57%, 38%, 32%, 30%, 42%, 33%	Astrocytoma (81%, 71%, 56%, 70%, 67%, 79%), Oligodendroglioma (14%, 24%, 35%, 26%, 33%, 12%), Oligoastrocytoma (5%, 5%, 9%, 4%, 0%, 9%)	*Location:* frontal (33%, 43%, 68%, 33%, 58%, 34%), parietal, occipital (24%, 24%, 9%, 29%, 0%, 30%), temporal (19%, 19%, 23%, 38%, 38%, 30%), deep structures (14%, 9%, 0%, 0%, 0%, 0%), other (10%, 5%, 0%, 0%, 4: 6%); *Laterality:* right (52%, 29%, 47%, 52%, 50%, 39%), left (43%, 62%, 53%, 48%, 42%, 58%), bilateral (5%, 10%, 0%, 0%, 8%, 3%)	Surgery: biopsy (38%, 33%, 52%, 57%, 33%, 55%); resection (62%, 67%, 48%, 43%, 67%, 45%); Radiotherapy (57%, 57%, 56%, 61%, 42%, 58%)	*Since diagnosis:* > 1 year; *Since treatment:* > 1 year
Leonetti (2021) (Italy) [45]	80	39.7 (11.3)	42.5%	Astrocytoma (30%), Oligodendroglioma (28.75%), Gangoglioma (17.5%), Other (23.75%)	*Location:* frontal (51.2%), insular (18.2%), temporal (12.5%), parietal (16.3%), other (1.3%); *Laterality:* right (53.8%), left (46.3%)	Surgery (100%), Radiotherapy (52.5%), Chemotherapy (57.5%)	From point of diagnosis to 12 months post-surgery
Mahalakshmi (2015) (India) [46]	54	NR^h^	NR^h^	NR^h^	NR^h^	NR	*Since diagnosis:* 3 months
Okita (2015) (Japan) [47]	50	Median 39 (22–76)	32%	Astrocytoma (72%), Oligodendroglioma (6%), Oligoastrocytoma (22%)	*Location:* frontal (47.6%, 52.6%, 60%); temporal (19.1%, 36.8%, 10%); parietal (23.8%, 0%, 20%)^d^	Radiotherapy (78%), Chemotherapy (60%)	*Since treatment:* 0–4 years (*n* = 21), 5–9 years (*n* = 19), 10–20 years (*n* = 10); median 5.8 years (0–20.2 years)
Reijneveld (2001) (Netherlands) [48]	24	38.2 (10.6)	37.5%	NR	*Location:* frontal (*n* = 4), temporal (*n* = 7), parietal (*n* = 7), occipital (*n* = 3), midline (*n* = 3)	Stereotactic biopsy (*n* = 6), surgery (*n* = 17), unknown (*n* = 1)	*Since diagnosis:* mean 5.5 years (SD 3.7 years)
Ruge (2011) (Germany) [49]	33	44.4 (11.2)	51.5%	Astrocytoma (90.9%), Oligodendroglioma (6.1%), Oligoastrocytoma (3.0%)	*Location:* frontal (33.3%), temporal (54.5%), parietal (6%), subcortical (6%); *Laterality:* left (55.5%), Right (45.5%)	NR	NR
Salo (2002); Mainio (2006) (Finland) [50, 51]	19	Male: 49.4 (12.9) Female: 48.8 (13.7)	52.6%	NR	NR	Surgery 100%	NR
Teng (2021) (Australia) [52]	167; 1: 64, 2: 51, 3: 25, 4 + : 27^e^	40.85 (13.47)	46.1%	Grade II diffuse glioma (86.23%), Grade I pilocytic astrocytoma (9.58%), other (4.19%); Grade I (11.98%), Grade II (88.02%)	*Laterality:* right (47.31%), left (40.72%), midline (7.78%), unknown (4.19%)	Resection: biopsy (19.16%), partial (7.19%), subtotal (36.53%), gross-macroscopic (29.34%), unknown (7.78%); Radiotherapy (31.14%), Chemotherapy (13.17%)	*Since treatment:* mean 60.66 (SD 110.48) months
Umezaki (2020) (Japan) [53]	31	NR^h^	NR^h^	Diffuse astrocytoma (*n* = 11), Oligodendroglioma (*n* = 16), Oligoastrocytoma (*n* = 3), Diffuse glioma (*n* = 1)	NR^h^	NR^h^	NR^h^
Wang (2018); Li (2019a; 2019b) (China) [54–56]	260	Median 42 (18–67)	44.20%	Grade I (23.5%), Grade II (76.5%)	*Laterality:* left (45.8%), right (52.7%), bilateral (1.5%)	Excision: total (48.8%), subtotal (43.5%), biopsy (7.7%); Surgery (33.5%), surgery and radiotherapy (57.3%), surgery and chemotherapy (3.8%), surgery, radiotherapy, and chemotherapy (5.4%)	*Since treatment:* 1 month
Yavas (2012) (Turkey) [57]	43; T1: 43, T2: 43, T3: 42, T4: 41, T5: 39, T6: 37, T7: 30, T8: 21^f^	18–29 (20.93%), 30–39 (39.53%), 40–49 (20.93%), 50–59 (13.95%), 60–69 (4.65%)	37.2%	Grade I (9.3%), Grade II (81.4%), Not other classified low grade (9.3%)	*Location:* frontal (48.8%), parietal (16.3%), temporal (27.9%), occipital (7%); *Laterality:* left (41.9%), right (58.1%)	Excision: total (23.3%), subtotal (65.1%), inoperative (11.6%); Radiotherapy (100%)	*Since treatment:* initial assessment at end of radiotherapy

*NR* = *Not reported*

^a^T1 = mean 6 years, T2 = mean 12 years since diagnosis

^b^T1 = baseline, T2 = 6 months, T3 = 12 months follow-up

^c^Sample stratified into six levels of epilepsy burden (level 1 = epilepsy free – level 6 =  > 6 seizures in the last year)

^d^Stratified by: Years since treatment 0–4, 5–9, 10–20

^e^Number of surveys completed by participants within the sample

^f^T1 = baseline, T2 = 1–3 months, T3 = 6 months, T4 = 12 months, T5 = 18 months, T6 = 24 months, T7 = 30 months, T8 = 36 months since treatment

^g^Where mean age was not reported, medians and age groups are detailed

^h^This demographic was not reported separately for low-grade gliomas

Tumour details included: grade (*n* = 5 studies) [31, 43, 52, 54, 57]; type (*n* = 12) [31, 33, 36, 40–42, 44, 45, 47, 49, 52, 53], predominantly astrocytoma, oligodendroglioma, and oligoastrocytoma; laterality (*n* = 13) [31, 33, 35, 36, 38, 39, 42, 44, 45, 49, 52, 54, 57]; and location (*n* = 12) [31, 33, 35, 36, 39, 42, 44, 45, 47–49, 57], largely frontal lobe. Four studies reported genetic markers [33, 42, 45, 53]. Sixteen studies reported treatment [12, 33, 35, 36, 38, 40–42, 44, 45, 47, 48, 50, 52, 54, 57], including chemotherapy, radiotherapy, and extent of surgical resection. Time since diagnosis/treatment at which HRQoL was assessed ranged from point of diagnosis to 20-years since treatment. Heterogeneity was common within studies; e.g. participants in Correa et al. [36] ranged from six- to 118-months (9.83 years) since treatment.

### Study design

Thirteen studies were cross-sectional [34, 35, 39–44, 46–49, 53] and nine longitudinal [31, 33, 37, 38, 45, 51, 52, 54, 57], assessing HRQoL at several (albeit varied) timepoints (*Table **2*). Thirteen studies included a comparator and/or control group, comprising: HGG patients (*n* = 7) [34, 38, 39, 43, 45, 46, 53], non-cancer controls (NCC) (*n* = 6) [12, 35, 44, 48, 49, 52], or benign brain tumour [34], suspected LGG [48], or non-Hodgkin’s lymphoma (NHL)/chronic lymphocytic leukaemia (CLL) patients [12].

**Table 2:** Study characteristics of included quantitative studies that assessed health-related quality of life in low-grade glioma patients

Study	Quality appraisal (score)^a^	Study design	Comparator/ control	Measurement time points	HRQoL instrument(s) used	Factors examined for association with HRQoL
Aaronson (2011); Boele (2014; 2015); Ediebah (2017) [12, 30–32]	Good (21); Good (18); Good (21); Good (19)	Longitudinal	Non-Hodgkin’s lymphoma, chronic lymphocytic leukaemia, non-cancer controls	T1 = mean 6 years, T2 = mean 12 years since diagnosis	SF-36, BN20	Age, cognitive function, education, epilepsy burden, sex, time since diagnosis, treatment, tumour location
Affronti (2018) [33]	Acceptable (15)	Longitudinal	NR	T1 = 2 months, T2 = 4 months, T3 = 6 months since diagnosis	FACT-Br, FACIT- fatigue, FACT-Cog	Genetic markers
Budrukkar (2009) [34]	Acceptable (16)	Cross-sectional	High-grade glioma, benign tumour	Single time point (pre-adjuvant therapy)	QLQ-C30, BN20	Age, education, KPS, sex, socio-economic status, treatment, tumour location
Campanella (2017) [35]	Good (18)	Cross-sectional	Non-cancer controls	Single time point (> 1-year post-surgery, mean 3.35 years)	PWB	Age, cognitive function, education, epilepsy burden, sex, time since treatment, treatment, tumour location
Correa (2007; 2008) [36, 37]	Acceptable (15); Acceptable (15)	Longitudinal	NR	T1 = Baseline, T2 = 6 months, T3 = 12 months follow-up	FACT-Br	NR
Drewes (2018) [38]	Good (21)	Longitudinal	High-grade glioma	T1 = initial assessment (1–3 days before first surgery) T2 = 1 month, T3 = 6 months since treatment	EQ-5D	Treatment
Gabel (2019) [39]	Good (17)	Cross-sectional	High-grade glioma	Single time point (at diagnosis and initial clinic visit)	NIH-PROMIS, Neuro-QoL	NR^b^
Gustafsson (2006) [40]	Good (17)	Cross-sectional	NR	Single time point (mean 16 years since diagnosis)	QLQ-C30	Age, coping, marital status, sex, time since diagnosis^c^
Jakola (2012) [41]	Good (17)	Cross-sectional	NR	Single time point (mean 7 years since treatment)	QLQ-C30, BN20, EQ-5D	Tumour location
Jiang (2019) [42]	Good (17)	Cross-sectional	NR	Single time point (3 months since treatment)	SF-36	Post-traumatic stress disorder
Kim (2020) [43]	Good (17)	Cross-sectional	High-grade glioma	Single time point (not specified)	FACT-G	NR^b^
Klein (2003) [44]	Good (18)	Cross-sectional	Non-cancer controls	Single time point (not specified)	SF-36	Epilepsy burden
Leonetti (2021) [45]	Good (18)	Longitudinal	High-grade glioma	T0 = 1 week pre-surgery, T1 = 1 month, T2 = 3 months, T3 = 6 months, T4 = 12 months since surgery	SF-36	Age, cognitive function, education, genetic markers, sex, treatment, tumour location
Mahalakshmi (2015) [46]	Good (19)	Cross-sectional	High-grade glioma	Single time point (3 months since diagnosis)	QLQ-C30, BN20	NR^b^
Okita (2015) [47]	Good (17)	Cross-sectional	NR	Single time point (G1: 0–4; G2: 5–9; G3: 10–20 years since treatment)	QLQ-C30, BN20	Age, history of recurrence, KPS, time since treatment, treatment
Reijneveld (2001) [48]	Acceptable (15)	Cross-sectional	Suspected low-grade glioma, non-cancer controls	Single time point (> 6 months since diagnosis)	SF-36, BN20	NR
Ruge (2011) [49]	Good (19)	Cross-sectional	Non-cancer controls	Single time point (at diagnosis)	SF-36	Age, cognitive function, depression, duration of symptoms, KPS, seizures, tumour location
Salo (2002); Mainio (2006) [50, 51]	Acceptable (16); Acceptable (15)	Longitudinal	NR	T1 = Pre-surgery, T2 = 1 year, T3 = 5 years post-surgery	Sin-tonen’s 15D Nottingham Health Profile	NR
Teng (2021) [52]	Acceptable (16)	Longitudinal^d^	Non-cancer controls	Multiple timepoints completed at 6 monthly intervals, stratified by time since surgery and divided into 12 monthly intervals	QLQ-C30	Time since treatment^c^
Umezaki (2020) [53]	Good (19)	Cross-sectional	High-grade glioma	Single time point (not specified)	QLQ-C30, BN20	NR^c^
Wang (2018); Li (2019a; 2019b) [54–56]	Good (21); Good (20); Good (20)	Longitudinal	NR	T1 = 1 month, T2 = 1 year post- surgery	FACT-Br	Age, coping, depression, marital status, post-traumatic growth, post-traumatic stress disorder, seizures, sex, socio-economic status, time since treatment, treatment, tumour location, tumour type
Yavas (2012) [57]	Acceptable (15)	Longitudinal	NR	T1 = pre-adjuvant therapy, T2 = 1–3 months, T3 = 6 months, T4 = 12 months, T5 = 18 months, T6 = 24 months, T7 = 30 months, T8 = 36 months since treatment	QLQ-C30, BN20	Treatment

*BN-20* European Organisation for Research and Treatment of Cancer Quality of life Questionnaire Brain Neoplasm, *EQ-5D* EuroQoL 5 dimension, *FACT-Br* Functional Assessment of Cancer Therapy – Brain, *FACT-Cog* Functional Assessment of Cancer Therapy – Cognitive function, *FACT-G* Functional Assessment of Cancer Therapy – General, *FACIT-Fatigue* Functional Assessment of Chronic Illness Therapy – Fatigue, *G* Group, *NIH-PROMIS* National Institutes of Health—Patient-Reported Outcomes Measurement Information System, *Neuro-QoL* Quality of Life in Neurological Disorders, *NR* Not reported, *PWB* Psychological wellbeing scale, *QLQ-C30* European Organisation for Research and Treatment of Cancer Quality of Life Questionnaire Core, *SF-36* Short Form 36 health survey questionnaire, *T* Timepoint

^a^Where more than one paper reports the same study, quality appraisal scores are given for each individual paper

^b^Factors were not examined separately in low-grade gliomas

^c^Health-related quality-of-life patient reported outcome measures were examined as factors

^d^Participants could enter the study cohort at any point during follow-up and were then followed over time

Thirteen different general, cancer-related, brain tumour-specific, or unidimensional HRQoL instruments were used, predominantly: EORTC QLQ-BN20 (*n* = 8) [12, 34, 41, 46–48, 53, 57], EORTC QLQ-C30 (*n* = 8) [34, 40, 41, 46, 47, 52, 53, 57], SF-36 (*n* = 6) [12, 42, 44, 45, 48, 49], FACT-Br (*n* = 3) [33, 36, 54], and EQ-5D (*n* = 2) [38, 41]. Eleven studies used multiple HRQoL instruments [12, 33–35, 39, 41, 46–48, 50, 53, 57], often combining general (e.g. SF-36) or cancer-related (e.g. QLQ-C30), with brain tumour-specific (e.g. QLQ-BN20) instruments. The HRQoL instruments used, specific dimensions assessed by each, and their scoring, is detailed in Supplementary Table S4. Fourteen studies assessed global HRQoL [33, 34, 36, 38, 40, 41, 43, 46, 47, 51–54, 57] with one of the six instruments (e.g. FACT-G) with a possible global HRQoL score. Once grouped, frequently assessed HRQoL dimensions included: physical (*n* = 19 studies) [12, 33, 34, 36, 39–49, 52–54, 57], social (*n* = 18) [12, 33, 34, 36, 40–49, 52–54, 57], emotional (*n* = 13) [33, 34, 36, 39–41, 43, 46, 47, 52–54, 57], and cognitive functioning (*n* = 10) [33, 39–41, 46, 47, 52, 53, 57], as well as pain (*n* = 17) [12, 34, 38–42, 44–50, 52, 53, 57], and fatigue (*n* = 10) [33, 34, 39–41, 46, 47, 52, 53, 57].

### Quality appraisal

Quality appraisal scores ranged from 15 to 21 of a possible 24, with 20 papers considered ‘*good quality*’ [12, 30–32, 35, 38–47, 49, 53–56] and nine ‘*acceptable quality*’ [33, 34, 36, 37, 48, 50–52, 57] (Table 2; Supplementary Table S5). Aaronson et al. [12], Boele et al. [31], Drewes et al. [38], and Wang et al. [54] were the highest quality papers, each scoring 21. Primary reasons for lower scores included: failure to clearly document participant eligibility and recruitment (e.g. 11 papers (eight studies) excluded cognitively and/or communication impaired patients without detailing how this was determined) [12, 30–32, 38, 40, 42, 44, 46, 53, 57]; and lack of a control and/or comparator group.

### Health-related quality-of-life findings

#### Health-related quality-of-life

The dimensions measured and how scores are determined across the 11 multidimensional and two unidimensional HRQoL instruments reported in the studies is quite different (Table 2; Supplementary Table S4). HRQoL values were not reported for all potential instrument dimensions in 13 studies [34–36, 41–46, 48–50, 52]; below, the denominator is the number of studies that reported a value for a specific dimension.

#### Global HRQoL

Thirteen (of 14) studies reported poor global HRQoL in LGG patients (i.e. QLQ-C30 score 61.9–74) [33, 34, 36, 38, 40, 41, 43, 46, 47, 52–54, 57] that was significantly worse than in NCCs (*n* = 1 of one) [52], though significantly better than in HGG patients (*n* = 4 of five) [34, 38, 43, 46] (Table 3; Supplementary data).

**Table 3:** Health-related quality-of-life key findings

Study	Global HRQoL^a^	Specific HRQoL	HRQoL over time^a^	Findings vs comparators^a^
Aaronson (2011); Boele (2014; 2015); Ediebah (2017) [12, 30–32]	NA	*Function:* poor general health perception, mental health, physical role functioning, physical and mental components scores, and vitality *Symptom:* high levels of communication deficit, drowsiness, future uncertainty, and suffering from headaches	LGG patients had significantly worse physical component scores (*P* < 0.01) and physical functioning (*P* < 0.01) at long-term follow-up (mean 12 years) compared to mid-term follow-up (mean 5.6 years since diagnosis). No other significant differences were observed. Authors state that most LGG patients maintained a stable level of HRQoL	Compared with NCCs, LGG patients had significantly less bodily pain (*P* < 0.01), but significantly worse emotional role functioning, general health perception, mental component score, physical functioning, physical role functioning, social functioning, vitality (all *P* < 0.01), and mental health (*P* = 0.043). No significant differences between LGG patients and NHL/CLL comparators were observed
Affronti (2018) [33]	Poor	*Function:* poor brain cancer subscale scores, emotional and functional wellbeing. High perceived cognitive impairments, impact of perceived cognitive function, and poor perceived cognitive abilities *Symptom:* high levels of fatigue	LGG patients with either IDH mut or TERT mut (genetic markers) consistently reported lower global HRQoL, and higher levels of fatigue, depression, and distress from two to six months since diagnosis. *No significant differences as this was a pilot study*	NA
Budrukkar (2009) [34]	Poor	*Function:* poor cognitive, emotional, and social functioning *Symptom:* high levels of appetite loss, communication deficit, fatigue, insomnia, motor dysfunction, nausea/vomiting, pain, seizures, and suffering from headaches	NA	Compared with HGGs, LGG patients reported significantly better global HRQoL (*P* = 0.015). No significant differences between LGG patients and benign tumour comparators were observed
Campanella (2017) [35]	NA	*Function:* average psychological wellbeing	NA	Compared with NCCs, LGG patients scored significantly higher on the environmental mastery subscale of psychological wellbeing (*P* < 0.01)
Correa (2007; 2008) [36, 37]	Poor	NR	No significant changes over time in FACT-Br were observed	NA
Drewes (2018) [38]	Poor	NR	No significant differences in EQ-5D index scores were observed between all three time points	Compared with HGGs, LGG patients scored significantly higher on the EQ-5D at six months since treatment (*P* < 0.01)
Gabel (2019) [39]	NA	*Function:* high Neuro-QoL cognitive function and PROMIS physical function impairments *Symptom:* high PROMIS pain interference and sleep disturbance impairment	NA	Compared with HGGs, LGG patients experienced significantly greater distress related to pain intensity (*P* = 0.01) and declining physical function (*P* = 0.05)
Gustafsson (2006) [40]	Poor	*Function:* poor cognitive, emotional, and role functioning *Symptom:* high levels of dyspnoea, fatigue, financial difficulties, insomnia, and pain	NA	NA
Jakola (2012) [41]	Poor	*Function:* poor cognitive functioning *Symptom:* high levels of communication deficit, fatigue, future uncertainty, and motor dysfunction	NA	NA
Jiang (2019) [42]	NA	*Function:* poor emotional and physical role functioning, general health perception, mental health, physical functioning, social functioning, and vitality *Symptom:* high levels of bodily pain All eight dimensions were significantly worse in LGG patients with PTSD	NA	NA
Kim (2020) [43]	Poor	NR	NA	Compared with HGGs, LGG patients had significantly better global HRQoL (*P* < 0.01)
Klein (2003) [44]	NA	*Function:* poor physical and mental component scores that were increasingly worse in those with greater epilepsy burden	NA	Compared with NCCs, LGG patients had significantly more bodily pain, and worse emotional and physical role functioning, general health perception, physical functioning, vitality, (all *P* < 0.01), and social functioning (*P* = 0.013)
Leonetti (2021) [45]	NA	*Function:* high prevalence of poor physical and mental component scores	The prevalence of LGG patients with low SF-36 mental and physical component scores reduced incrementally from pre-surgery to one-year post-surgery, though statistical tests were not reported	No significant differences between LGG patients and HGG comparators were observed
Mahalakshmi (2015) [46]	Poor	*Symptom:* high levels of appetite loss, communication deficit, dyspnoea, fatigue, future uncertainty, motor dysfunction, nausea/vomiting, and very high financial difficulties	NA	Compared with HGGs, LGG patients had significantly better global HRQoL (*P* = 0.04), emotional functioning (*P* = 0.05), physical functioning (*P* < 0.01), role functioning (*P* = 0.01), and social functioning (*P* < 0.01). LGG patients also had significantly lower levels of communication deficit (*P* = 0.02), distress from hair loss (*P* = 0.05), fatigue (*P* < 0.01), nausea/vomiting (*P* = 0.05), pain (*P* = 0.01), seizures (*P* = 0.01), and suffering from headaches (*P* = 0.04), though greater financial difficulties (*P* = 0.02) than HGG patients
Okita (2015) [47]	Poor	*Symptom:* high levels of communication deficit, difficulty with bladder control, drowsiness, fatigue, financial difficulties, future uncertainty, suffering from headaches, and weakness of legs	NA	NA
Reijneveld (2001) [48]	NA	*Function:* poor general health perception, emotional and physical role functioning, mental health, and vitality *Symptom:* high levels of communication deficit, future uncertainty, seizures, and suffering from headaches	NA	Compared with NCCs, LGG patients scored significantly worse on general health perception, mental health, social functioning (all *P* < 0.05), and vitality (*P* < 0.01). LGG patients also scored significantly worse than suspected LGGs on vitality (*P* < 0.05) and had higher levels of difficulty with bladder control (*P* < 0.05) and motor dysfunction (*P* < 0.01)
Ruge (2011) [49]	NA	*Function:* poor general health perception, mental health, and vitality	NA	Compared with NCCs, LGG patients scored significantly worse on general health perception, emotional role functioning, mental health, social functioning (all *P* < 0.01), and physical role functioning (*P* < 0.025)
Salo (2002); Mainio (2006) [50, 51]	Average	NR	NR	NA
Teng (2021) [52]	Poor	*Function:* poor cognitive, emotional, and social functioning	Conclude that LGG patients sustain clinically significant impairments to global HRQoL, particularly cognitive, emotional, role, and social functioning, as well as high levels of fatigue and insomnia at 12-month intervals across 10 years	Compared with NCCs, LGG patients reported significantly worse global HRQoL, cognitive, emotional, physical, role, and social functioning (all *P* < 0.01)
Umezaki (2020) [53]	Poor	*Symptom:* high levels of communication deficit, drowsiness, fatigue, financial difficulties, future uncertainty, and weakness of legs	NA	Compared with HGGs, LGG patients reported significantly lower levels of constipation (*P* = 0.04), distress from hair loss (*P* = 0.02) and itchy skin (*P* = 0.04). No significant differences between LGG patients and HGG comparators were observed in all functioning domains assessed
Wang (2018); Li (2019a; 2019b) [54–56]	Poor	*Function:* poor brain cancer subscale scores, emotional and functional wellbeing	HRQoL was significantly better at one-year post-surgery than one-month post-surgery for emotional wellbeing, functional wellbeing, brain tumour subscale, and global HRQoL (all *P* < 0.01)	NA
Yavas (2012) [57]	Poor	*Function:* poor cognitive functioning *Symptom:* high levels of drowsiness, distress from hair loss, fatigue, financial difficulties, insomnia, and suffering from headaches	From initial assessment (*end of radiotherapy*) to 3 years since treatment, there were significant improvements in global HRQoL scores, future uncertainty, communication deficit, suffering from headaches, drowsiness, and distress from hair loss (all *P* < 0.01). More specifically, future uncertainty significantly improved from initial assessment to 2 years and 3 years (both *P* < 0.01), but not 1 year. There was progressive improvement in communication deficit across follow-up, but only significant between initial assessment and 3 years (*P* = 0.016). Compared to initial assessment, there were significant improvements to suffering from headaches at 2 and 3 years (both *P* < 0.01), but not 1 year. There were significant improvements to drowsiness from initial assessment to 1, 2, and 3 years (all *P* < 0.01). Distress from hair loss was significantly worse at 1 year, than initial assessment (*P* = 0.01), but not at 2 or 3 years. No other significant differences were observed	NA

*HGG* High-grade glioma, *HRQoL* Health-related quality-of-life, *LGG* Low-grade glioma; *NA* not assessed, *NCC* non-cancer controls, *NHL/CLL* non-Hodgkin’s lymphoma/chronic lymphocytic leukaemia, *NR* not reported

^a^Key findings were ‘not assessed’ if the instrument used did not determine a global HRQoL score, a comparator/control was not included, or the study was cross-sectional and only measured a single time point

#### Specific HRQoL – functioning

Seventeen studies reported values for functioning aspects of HRQoL in LGG patients [12, 33, 34, 39–42, 44–49, 52–54, 57]. Poor functioning was reported across numerous HRQoL aspects. Cognitive functioning was poor in seven (of 10) studies [33, 34, 39–41, 52, 57], and significantly worse than NCCs in one (of one) of these [52]. Poor emotional functioning was reported in five (of 11) studies [33, 34, 40, 52, 54] and was significantly worse than NCCs in one (of one) of these [52]. General health perception was poor in four (of five) studies [12, 42, 48, 49]; four (of four studies with an NCC group) found it was significantly worse in LGG patients than in NCCs [12, 44, 48, 49]. Poor vitality was reported in four (of five) studies [12, 42, 48, 49]; three (of four studies with an NCC group) found it was significantly worse than in NCCs [12, 44, 48], as well as suspected LGGs in one (of one) of these [48].

Compared to NCCs, studies also reported significantly worse physical functioning (*n* = 4 of five) [12, 44, 49, 52] and emotional role functioning (*n* = 3 of four) [12, 44, 49] in LGG patients. Compared to HGG patients, of seven studies, only Mahalakshmi et al. [46] found significant differences, namely that LGG patients reported better emotional, physical, and social functioning.

Across studies, functioning aspects with the worst scores were cognitive functioning (*n* = 6) [39, 41, 46, 47, 52, 57], functional wellbeing (*n* = 2) [33, 54], general health perception (*n* = 2) [42, 49], social functioning (*n* = 2) [34, 53], vitality (*n* = 2) [12, 48], role functioning [40], and SF-36 mental [44] and physical [45] component scores. Still, ‘*worst scores*’ is a function (in part) of instrument used and what study authors choose to report, as cognitive functioning was either not assessed or reported in seven of the 11 studies that reported another aspect as having the worst score.

#### Specific HRQoL – symptoms

Fourteen studies reported values for HRQoL symptoms [12, 33, 34, 39–42, 46–50, 53, 57]. Considerable symptom burden was evident, most notably high levels of fatigue (reported in *n* = 8 of nine studies) [33, 34, 40, 41, 46, 47, 53, 57]. Other symptoms with substantial burden included: communication deficits (*n* = 7 of eight) [12, 34, 41, 46–48, 53]; future uncertainty (*n* = 6 of eight) [12, 41, 46–48, 53]; suffering from headaches (*n* = 5 of seven) [31, 34, 47, 48, 57]; financial difficulties (*n* = 5 of five) [40, 46, 47, 53, 57]; drowsiness (*n* = 4 of seven) [31, 47, 53, 57]; insomnia (*n* = 4 of five) [34, 39, 40, 57]; pain (*n* = 4 of 12) [34, 39, 40, 42]; and motor dysfunction (*n* = 3 of eight) [34, 41, 46]. The two studies that compared pain in LGG patients with NCCs were inconsistent [12, 44]. One study found motor dysfunction was significantly worse in LGG patients than those with suspected LGGs [48].

Again, compared to HGG patients, significant differences were primarily reported by Mahalakshmi et al. [46]; LGG patients had lower levels of communication deficit, distress from hair loss, fatigue, nausea/vomiting, pain, seizures, and suffering from headaches, though greater financial difficulties.

Across studies, symptoms with the worst scores were fatigue (*n* = 6) [33, 34, 40, 41, 47, 57], sleep disturbances (*n* = 2) [39, 50], drowsiness [53], financial difficulties [46], future uncertainty [12], and seizures [48]. This may be influenced by instrument used, as fatigue (*n* = 10) was the second most assessed symptom.

### Health-related quality-of-life over time

Longitudinal studies varied in the timepoints at which they measured HRQoL. Four of nine longitudinal studies (which considered different aspects of HRQoL) suggested HRQoL remains low, but stable, over time, specifically over six-months [38], one-year [37], and up to 10-[52] and 12-years since diagnosis or treatment [31] (Table 3).

#### Global HRQoL changes

In Wang et al. [54] and Yavas et al. [57], global HRQoL improvements were reported over one- and three-years since treatment, respectively. For Yavas et al. [57], the median improvement was consistent with the EORTC QLQ-C30 definition of a minimally important difference (i.e. 4–6 points) for global HRQoL improvement in glioma patients [58].

#### Specific HRQoL changes

For Wang et al., emotional and functional wellbeing, and FACT-Br brain tumour subscale scores significantly improved at one-year, compared to one-month since treatment [54]. In Yavas et al., future uncertainty, communication deficit, suffering from headaches, drowsiness, and distress from hair loss significantly improved from initial assessment (*end of radiotherapy*) to three-years since treatment [57]. For Boele et al., with longer term follow-up, SF-36 physical functioning and physical component scores worsened between a mean of 5.6 and 12 years since diagnosis [31].

### Factors associated with health-related quality-of-life

Eighteen papers reporting 15 studies [12, 30, 33–35, 38, 40–42, 44, 45, 47, 49, 52, 54–57] examined 19 different factors for association with HRQoL, most often: age (*n* = 8 studies) [12, 34, 35, 40, 45, 47, 49, 54], treatment (*n* = 8) [12, 34, 35, 38, 45, 47, 54, 57], and tumour location (*n* = 7) [12, 34, 35, 41, 45, 49, 54]. Significant associations were observed by at least one study for 17 factors (i.e. all except genetic markers and marital status) (Table 4). For eight factors—age, cognitive function, education, sex, SES, time since diagnosis/treatment, treatment, and tumour location—reported associations were not always statistically significant; the remaining nine factors – coping, depression, duration of symptoms, epilepsy/seizure burden, history of recurrence, KPS, post-traumatic growth, post-traumatic stress disorder (PTSD), and tumour type—were significantly associated with HRQoL in all studies in which they were reported.

**Table 4:** Factors associated with health-related quality-of-life

Factor	Paper	Finding
Age^*^	Aaronson (2011) [12]	Older age was significantly associated with worse visual disorder (*P* = 0.039)
	Budrukkar (2009) [34]	No significant associations were observed
	Campanella (2017) [35]	No significant associations were observed
	Gustafsson (2006) [40]	No significant associations were observed
	Leonetti (2021) [45]	No significant associations were observed
	Okita (2015) [47]	Older age (≥ 40) was significantly associated with lower levels of diarrhoea (*P* = 0.05)
	Ruge (2011) [49]	No significant associations were observed
	Wang (2018); Li (2019) [54, 56]^a^	No significant associations were observed
Cognitive function^*^	Boele (2014) [30]	Greater executive functioning, processing speed, verbal memory, working memory, information processing, and attention were significantly associated with lower levels of future uncertainty (all *P* < 0.01), visual disorder (all *P* < 0.01; verbal memory *P* = 0.011), motor dysfunction (all *P* < 0.01), communication deficit (*P* < 0.01; verbal memory *P* = 0.011; executive functioning *P* = 0.034; processing speed not significant), and less seizures (all *P* < 0.01), and drowsiness (processing speed and information processing *P* < 0.01; executive functioning *P* = 0.014; verbal memory *P* = 0.029; working memory *P* = 0.011; attention not significant). Greater processing speed was significantly associated with more suffering from headaches (*P* = 0.018), while greater verbal memory (*P* = 0.044), working memory (*P* = 0.036), and information processing (*P* = 0.018) were significantly associated with less suffering from headaches
	Campanella (2017) [35]	No significant associations were observed
	Leonetti (2021) [45]	Higher levels of language deficit were significantly associated with worse mental component scores at 6-months (*P* = 0.014) and 1-year post-surgery (*P* < 0.01), and worse physical component scores at 3-months (*P* = 0.025), 6-months (*P* = 0.049), and 1-year post-surgery (*P* = 0.014)
	Ruge (2011) [49]	Better divided attention performance was significantly associated with better general health perception (*P* < 0.02) and less bodily pain (*P* < 0.05)
*Coping*^−^	Gustafsson (2006) [40]	Higher levels of avoidant coping were significantly associated with worse emotional functioning (*P* < 0.01). Higher confrontive coping was significantly associated with greater financial impact (*P* < 0.01) and worse role functioning (*P* < 0.01)
	Li (2019) [56]	Higher levels of avoidant coping were significantly associated with worse global HRQoL (*P* < 0.01)
*Depression*^−^	Ruge (2011) [49]	Higher levels of depression were significantly associated with worse vitality (*P* < 0.01), social functioning (*P* < 0.01), emotional functioning (*P* < 0.05) and mental health (*P* < 0.01)
	Wang (2018); Li (2019) [54, 55]^a^	Higher levels of depression were significantly associated with worse global HRQoL (*P* < 0.01)
*Duration of symptoms*^−^	Ruge (2011) [49]	Longer duration of symptoms (> 20 weeks) was associated with worse physical functioning (*P* = 0.043), vitality (*P* = 0.023), social functioning (*P* = 0.036), and emotional role functioning (*P* = 0.014)
*Education*^+^	Aaronson (2011) [12]	No significant associations were observed
	Budrukkar (2009) [34]	Higher level of literacy was significantly associated with better global HRQoL (*P* = 0.025)
	Campanella (2017) [35]	No significant associations were observed
	Leonetti (2021) [45]	No significant associations were observed
*Epilepsy/seizure burden*^−^	Aaronson (2011) [12]	Higher epilepsy burden was significantly associated with worse physical and mental component scores, and higher levels of future uncertainty, motor dysfunction, communication deficit, seizures (all *P* < 0.01), visual disorder (*P* = 0.019), suffering from headaches (*P* = 0.046), drowsiness (*P* = 0.033), and weakness of legs (*P* = 0.021)
	Campanella (2017) [35]	Higher epilepsy burden was significantly associated with worse psychological wellbeing (*P* = 0.013)
	Klein (2003) [44]	Higher epilepsy burden was significantly associated with worse physical health and mental component scores (both *P* < 0.01)
	Ruge (2011) [49]	Presence of seizures was significantly associated with worse social functioning (*P* < 0.05)
	Wang (2018); Li (2019) [54, 56]^a^	Presence of seizures was significantly associated with worse global HRQoL (*P* < 0.01)
*Genetic markers*	Affronti (2018) [33]	No significant associations were observed
	Leonetti (2021) [45]	No significant associations were observed
*History of recurrence*^−^	Okita (2015) [47]	A history of recurrence was significantly associated with worse cognitive functioning (*P* = 0.03) and higher levels of fatigue (*P* = 0.02), constipation (*P* = 0.01), financial difficulties (*P* = 0.01), visual disorder (*P* < 0.01), motor dysfunction (*P* = 0.04), communication deficit (*P* = 0.02), drowsiness (*P* = 0.02), weakness of legs (*P* = 0.01), and difficulty with bladder control (*P* = 0.02)
*KPS*^+^	Budrukkar (2009) [34]	Higher KPS was significantly associated with better global HRQoL (*P* = 0.04)
	Okita (2015) [47]	Higher KPS was significantly associated with better global HRQoL (*P* < 0.01), physical functioning (*P* < 0.01), role functioning (*P* = 0.03), and social functioning (*P* = 0.02), as well as lower levels of fatigue (*P* = 0.03), insomnia (*P* = 0.02), constipation (*P* = 0.01), motor dysfunction (*P* = 0.02), communication deficit (*P* = 0.02), drowsiness (*P* = 0.04), weakness of legs (*P* < 0.01), and difficulty with bladder control (*P* < 0.01)
	Ruge (2011) [49]	Higher KPS was significantly associated with better physical functioning (P < .01) and role functioning (P = .01)
*Marital status*	Gustafsson (2006) [40]	No significant associations were observed
	Wang (2018); Li (2019) [54, 56]^a^	No significant associations were observed
*Post-traumatic growth (PTG)*^+^	Wang (2018); Li (2019a; 2019b) [54–56]^a^	Higher PTG was significantly associated with better global HRQoL (*P* < 0.01)
*Post-traumatic stress disorder (PTSD)* ^−^	Jiang (2019) [42]	Those with PTSD had significantly worse HRQoL in all eight dimensions of the SF-36, than those without PTSD (*P* < 0.01; physical functioning: *P* = 0.026)
	Li (2019) [56]	Having PTSD was significantly associated with worse global HRQoL (*P* < 0.01)
*Sex*^−^	Aaronson (2011) [12]	Female sex was significantly associated with worse physical and mental component scores, and higher levels of visual disorder, motor dysfunction, suffering from headaches (all *P* < 0.01), and weakness of legs (*P* = 0.028)
	Budrukkar (2009) [34]	No significant associations were observed
	Campanella (2017) [35]	No significant associations were observed
	Gustafsson (2006) [40]	No significant associations were observed
	Leonetti (2021) [45]	No significant associations were observed
	Wang (2018); Li (2019) [54, 56]^a^	No significant associations were observed
*Socio-economic status (SES)*^+^	Budrukkar (2009) [34]	No significant associations were observed
	Wang (2018); Li (2019a; 2019b) [54–56]^a^	Having social insurance (*P* < 0.01) and higher SES (*P* < 0.01) were significantly associated with better global HRQoL
Time since diagnosis/ treatment^*^	Aaronson (2011) [12]	No significant associations were observed
	Campanella (2017) [35]	No significant associations were observed
	Gustafsson (2006) [40]	No significant associations were observed
	Okita (2015) [47]	Those 10–20 years since treatment had significantly more difficulty with bladder control than 0–4 years since treatment (*P* < 0.01)
	Teng (2021) [52]	Longer time since treatment was significantly associated with better role functioning (*P* = 0.013)
	Wang (2018); Li (2019a) [54, 55]^a^	Longer time since treatment was significantly associated with better global HRQoL (*P* < 0.01)
Treatment^*^	Aaronson (2011) [12]	Surgical intervention was significantly associated with higher levels of future uncertainty (*P* = 0.02). Radiotherapy was significantly associated with worse mental component scores (*P* = 0.029)
	Budrukkar (2009) [34]	No significant associations were observed
	Campanella (2017) [35]	No significant associations were observed
	Drewes (2018) [38]	No significant associations were observed
	Leonetti (2021) [45]	Receipt of adjuvant treatments was significantly associated with worse mental component scores at 6-months post-surgery (*P* < 0.01) and worse physical component scores at 3-months (*P* = 0.013) and 6-months post-surgery (*P* < 0.01)
	Okita (2015) [47]	Radiotherapy was significantly associated with lower levels of nausea and vomiting (*P* = 0.01) and dyspnoea (*P* = 0.04), but higher levels of communication deficit (*P* = 0.03). Chemotherapy was significantly associated with worse physical functioning (*P* = 0.05) and bladder control (*P* = 0.04)
	Wang (2018); Li (2019) [54, 56]^a^	No significant associations were observed
	Yavas (2012) [57]	No significant associations were observed
Tumour location^*^	Aaronson (2011) [12]	Tumour laterality was significantly associated with higher levels of communication deficit (*P* < 0.01) (*specific laterality not given*)
	Budrukkar (2009) [34]	No significant associations were observed
	Campanella (2017) [35]	No significant associations were observed
	Jakola (2012) [41]	No significant associations were observed
	Leonetti (2021) [45]	No significant associations were observed
	Ruge (2011) [49]	Temporal, parietal, and subcortical tumour locations were significantly associated with worse physical functioning (*P* = 0.014)
	Wang (2018) [54]	Right hemisphere location was significantly associated with better global HRQoL (*P* = 0.01)
*Tumour type*^+^	Li (2019) [56]	Lower tumour grade was significantly associated with better global HRQoL (*P* < 0.05)

*HRQoL* Health-related quality-of-life

^+^Positively associated

− Negatively associated

*Both positively and negatively associated

^a^The same finding was reported by more than one paper reporting the same study

There were 10 positively associated factors, with the most supporting evidence for KPS (*n* = 3 of three). Higher KPS was positively associated with global (*n* = 2) [34, 47] and specific HRQoL (e.g. less fatigue) (*n* = 2) [47, 49]. There were 12 negatively associated factors, with the most supporting evidence for epilepsy/seizure burden (*n* = 5 of five). Greater burden was negatively associated with global [54] and specific HRQoL (e.g. worse social functioning) (*n* = 4) [12, 35, 44, 49].

Five factors, namely, age, cognitive function, time since diagnosis/treatment, treatment, and tumour location were positively and negatively associated with HRQoL. For example, older age was positively [47] and negatively [12] associated with specific symptoms (e.g. diarrhoea and visual disorder, respectively).

## Discussion

### Summary of main findings

This systematic review aimed to identify quantitative evidence assessing HRQoL in adult LGG patients, to establish which aspects of HRQoL were impacted; how HRQoL compared with other populations; temporal trends; and factors associated with HRQoL. The 29 papers identified relating to 22 studies were largely good quality. Thirteen studies included comparator and/or control groups, and 19 factors were examined. Overall, the evidence-base suggests global HRQoL in LGG patients is poor, with considerable functioning impairments and symptom burden, most notably, cognitive functioning and fatigue, respectively. Over time, HRQoL remained low, but stable, and was better than in HGG patients, but substantially worse than in NCCs. Seventeen factors, most frequently epilepsy/seizure burden, were positively (*n* = 10 factors) or negatively (*n* = 12) associated with HRQoL.

### Health-related quality-of-life

Thirteen different HRQoL instruments were used. Given the variation across instruments and heterogeneity in patient samples and times at which HRQoL was assessed, we decided not to conduct a meta-analysis. We support Fountain et al.’s call, made in 2016, for a standardised set of validated HRQoL measures for future LGG studies [59]. However, since 2016, 11 studies in this review used 11 different instruments. Hence, this issue is ongoing and needs to be addressed.

Despite better HRQoL than in HGG patients, poor HRQoL in LGG patients was consistently reported, and was emphasised when compared to NCCs. Notable functioning impairments were observed for cognitive, emotional, physical role, and social functioning, general health perception, mental health, and vitality. Symptom burden was high for communication deficit, fatigue, future uncertainty, pain, and suffering from headaches. Cognitive functioning and fatigue were consistently the functioning aspect and symptom with the most impairment and burden, respectively.

Comparisons within LGG subtypes were not investigated in the eligible studies. Survival rates vary by subtype; 1–10 year survival is markedly higher in oligodendrogliomas (93.9 to 64%), than diffuse astrocytomas (72.2 to 37.6%) [3]. It is possible quality of survival also varies. Future research should compare HRQoL across LGG subtypes to distinguish whether impairments or symptoms are accentuated in particular tumour types. The EORTC QLQ-C30 reference values for brain tumours are worse than other cancers (i.e. breast and colorectal) [27]. However, research comparing HRQoL in LGG patients to other (non-brain) cancer populations is scarce. Such comparisons would be of value to help determine whether more tumour-specific, or targeted, supportive care services are required.

There was substantial heterogeneity in time since diagnosis/treatment at which HRQoL was assessed, from point of diagnosis to 20-years since treatment. In general, studies which included patients closer to diagnosis reported greater impacts on HRQoL, as sufficient time may not have elapsed to adjust. For example, in Jiang et al. [42], which included patients approximately 3-months post-diagnosis, SF-36 scores were considerably lower than in other studies. Assessing HRQoL in early stages post-diagnosis may also be problematic. Ruge et al. [49] abandoned the BN20 because LGG patients did not want to be prospectively confronted with questions about treatment effects and tumour progression.

### Health-related quality-of-life over time

There was considerable heterogeneity in timepoints assessed across longitudinal studies, with follow-up from one-month to 12-years since diagnosis. Post-treatment, HRQoL typically remained stable over time. However, largely poor baseline scores mean this is not an encouraging finding; rather it suggests LGG patients experience sustained HRQoL impairments over extended periods. Observed improvements to global and specific HRQoL were largely in comparison to one-month post-treatment and probably reflect dissipation of the more acute side-effects of adjuvant therapies [45]. Time for adjustment to the diagnosis is also important, and likely influences the temporal trends; acceptance has been associated with reduced general, and cancer-related, distress [60].

The longitudinal evidence is limited by failure to account for tumour progression or recurrence. Investigators tend not to make any accommodation in their results for the fact that some people have dropped out. Drewes et al. [38] gave deceased patients at follow-up a score of zero, which drove down their mean scores. In general, within these studies, observed temporal trends may, therefore, be biased by the dropout of those whose tumours have progressed and who might plausibly have worse HRQoL. This means more clarity is needed on how long HRQoL impairments are sustained, if, and when, they alleviate, and which aspects remain impaired over time.

### Factors associated with health-related quality-of-life

Eight factors were positively associated, while four factors were negatively associated, with global HRQoL. Five factors were positively associated, while 12 factors were negatively associated with specific aspects of HRQoL. Epilepsy/seizure burden was most consistently associated with worse HRQoL suggesting further seizure management, as a clinical priority, may ameliorate the impact of an LGG on patient HRQoL.

Eight factors had inconsistent findings, most notably, age, sex, treatment, and tumour location. Nonetheless, acknowledging these factors is important when considering what support may be needed. For example, PTSD was associated with worse global HRQoL [56], and worse functioning on all eight SF-36 dimensions [42]. Consequently, LGG patients with PTSD may benefit from enhanced supportive care.

### Critical appraisal of evidence

Twenty of the 29 papers were judged good quality. However, an important limitation is that the available evidence for HRQoL in adult LGG patients may not represent the full LGG population. Eleven studies explicitly excluded patients with communication and/or cognitive impairments. Only Drewes et al. [38] facilitated their inclusion, but this was through proxy ratings, which may not be reliable [61]. Eight studies failed to detail how impairments were determined [12, 38, 40, 42, 44, 46, 53, 57]. Gabel et al. [39] used the Boston Diagnostic Aphasia Examination [62], and Wang et al. [54] used the Mini Mental State Exam (MMSE) [63] to assess communication and cognitive impairments, respectively. Though indicative of impairment, this should not determine someone’s capacity to participate. Wang et al. [54] excluded patients with at least mild cognitive impairment (≤ 24), yet lower MMSE scores are significantly associated with worse HRQoL in brain tumour patients [64]. Therefore, the average HRQoL of LGG patients was likely overestimated.

Consistent with Brownsett et al. [65], we highlight the prevalence of poor cognitive functioning and high levels of communication deficit in adult LGG patients. However, explicit exclusion of patients with these impairments in over half of studies, means these impacts may be underestimated. For those that did not exclude such patients, if/how participation was facilitated was often unclear. Miscomprehension of a question due to such impairments could impact the reliability of results. Future research should do more to facilitate greater inclusivity. To achieve this, researchers might engage in supportive conversation training; ensure accessible formatting of study documentation; validate accessible (e.g. pictorial) rating scales (see the assessment for living with aphasia [66]); or involve/consult specialist professionals, such as speech and language therapists.

The WHO classification of tumours of the central nervous system was majorly restructured in 2016 [22], and 2021 [23]. Included studies were published 2001 to 2021, so what authors considered to be an LGG is potentially heterogeneous. Seven studies did not report tumour type [34, 35, 38, 39, 46, 48, 50], while three studies only reported tumour grade [43, 54, 57]. This may have implications for whether HRQoL findings accurately reflect LGG patients, as presently classified. Details of anti-cancer treatment(s), ethnicity and SES for study samples were also incompletely reported, which limits understanding of whether HRQoL vary by these factors. A minimum “core set” of socio-demographic, tumour, and treatment-related characteristics to be consistently reported by future study authors would be valuable.

## Strengths and limitations

Our review benefitted from extensive searches, including several databases and hand searching of reference lists and citations. Our focus on HRQoL beyond the clinical trial context allowed us to examine the ‘real world’ experience of LGG patients, when they are not undergoing the close monitoring that may happen within a trial.

A challenge was the lack of validated cut-off values for what is considered low, high, or clinically significant for numerous HRQoL instruments. Consequently, although we attempted to be consistent across studies, interpretation of reported values was difficult for some studies.

Brain tumour patients are likely to underestimate cognitive, emotional, psychological, and social changes [67]. This highlights an issue with subjective measurement of HRQoL using patient-reported outcome measures in LGG patients, namely that, because of the tumour, patients may lack insight and not self-report issues. This could mean functioning and symptoms have been over and underestimated, respectively, in the available studies.

## Future research

The international classification of functioning, disability, and health (ICF) has been used to consider 44 categories of activities and participation (e.g. walking or doing housework) that may be limited in primary brain tumour patients [68]. Future research could be conducted to understand whether, and if so, which HRQoL impairments, personal (e.g. age), clinical (e.g. tumour location), and environmental (e.g. location) factors are associated with these categories in LGG patients. This could help to further highlight specific support needs of this population overall, and subgroups within it. To do this, a useful first step would be to code the HRQoL instrument items to the ICF.

To date, one qualitative study has explored HRQoL in LGG patients [69], and this focussed largely on coping strategies used. Further qualitative research would be of value to provide a more holistic insight into patients’ experiences of HRQoL impacts, functional impairments, and symptoms, and how different impacts might be interconnected. Patients could reflect on when HRQoL aspects were particularly impacted, at what point these improved or deteriorated, and valuable (in)formal support.

## Conclusion

Influenced by several factors, most frequently, epilepsy/seizure burden, adult LGG patients have poor global HRQoL and experience an array of functioning impairments and symptom burden, most notably cognitive functioning and fatigue, respectively. These remain poor, but stable over time, and are markedly worse than in NCCs. Further consideration of LGG patients with speech, language, communication, and cognitive impairments is required, including steps to improve researchers’ confidence in ensuring their inclusion. These findings may help clinicians recognise current supportive care needs and inform types and timings of support needed, as well as inform future interventions.

## Supplementary Information

Below is the link to the electronic supplementary material.

Supplementary file1 (DOCX 596 kb)

Supplementary file2 (XLSX 37 kb)
