# Supplementary material for: Health-related quality of life in adults with low-grade gliomas: a systematic review
Source: Qual Life Res. 2022 Aug 6;32(3):625–51. doi: 10.1007/s11136-022-03207-x (PMC9992080; doi:10.1007/s11136-022-03207-x)
Supplement: Supplementary file 1 — Supplementary file1 (DOCX 596 kb) [file 11136_2022_3207_MOESM1_ESM.docx]

**Quality of life in adults with low-grade gliomas: a systematic review**

Ben Rimmer^*^, Iakov Bolnykh, Lizzie Dutton, Joanne Lewis, Richéal Burns, Pamela Gallagher, Sophie Williams, Vera Araújo-Soares, Fiona Menger, Linda Sharp

**Author affiliations:**

*Population Health Sciences Institute, Newcastle University, Newcastle University Centre for Cancer, Newcastle, England (B.R, L.D, V.A-S, L.S); Faculty of Medical Sciences, Newcastle University, Newcastle, England (I.B); Newcastle upon Tyne Hospitals NHS Foundation Trust, Newcastle, England (J.L, S.W); Department of Health and Nutritional Sciences, IT Sligo, Ireland (R.B); School of Psychology, Dublin City University, Dublin, Ireland (P.G); Faculty of Behavioural, Management and Social Sciences, Department of Health Technology and Services Research, University of Twente, The Netherlands (V.A-S); School of Education, Communication and Language Sciences, Newcastle University, Newcastle, England (F.M)*

**Corresponding author: Ben Rimmer, Population Health Sciences Institute, Newcastle University, Ridley Building 1, Newcastle upon Tyne, NE1 7RU.
Email: ben.rimmer@newcastle.ac.uk*

Contents

[Supplementary table 1: Search strategy 2](#_Toc101952492)

[Supplementary table 2: Database searches 3](#_Toc101952493)

[Supplementary table 3: Additional population characteristics 8](#_Toc101952494)

[Supplementary table 4: Quality-of-life instruments and their scoring 12](#_Toc101952495)

[Supplementary table 5: Quality appraisal 14](#_Toc101952496)

[Supplementary data: Overall quality-of-life findings 16](#_Toc101952497)

# Supplementary table 1: Search strategy

| **Area** | **Key terms** |
| --- | --- |
| *Low-grade glioma* | - Low* grade glioma* (or low-grade glioma* - for CINAHL) - LGG* - Anaplastic oligodendroglioma* - Oligodendroglioma/ - Astrocytoma/ - Diffuse astrocytoma* - Oligoastrocytoma* - Grade II astrocytoma* - Grade 2 astrocytoma* - Grade II oligodendroglioma* - Grade 2 oligodendroglioma* - Grade II oligoastrocytoma* - Grade 2 oligoastrocytoma* - Gemistocytic astrocytoma* - Pilomyxoid astrocytoma* |
| *Quality of life* | - Quality of life (EORTC QLQ-C30, BN-20, SF-36, FACT-G, FACT-Br*) - Health related quality of life (or Health-related quality of life – for CINAHL) - QoL - HRQoL |

# Supplementary table 2: Database searches


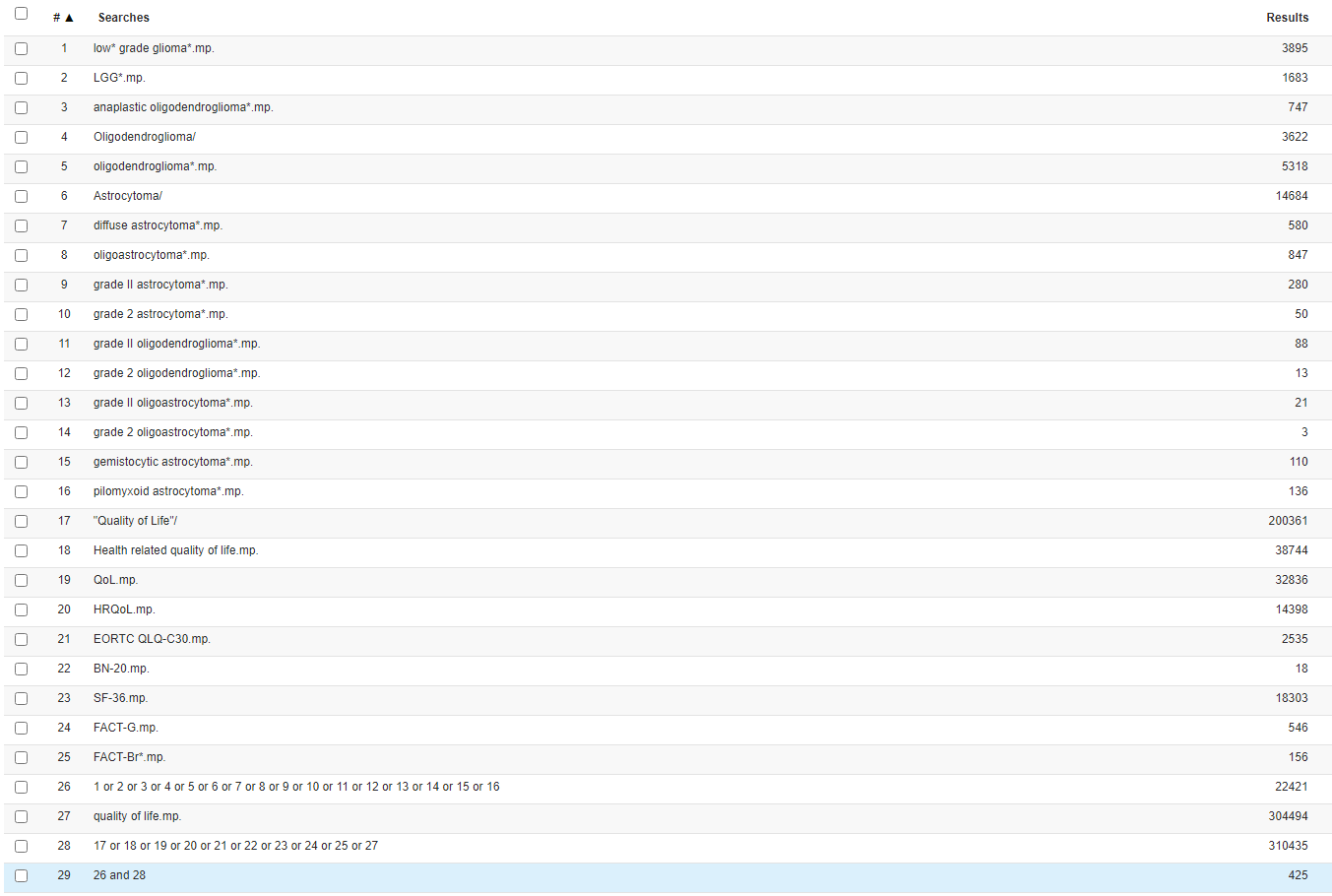
MEDLINE search

Embase search


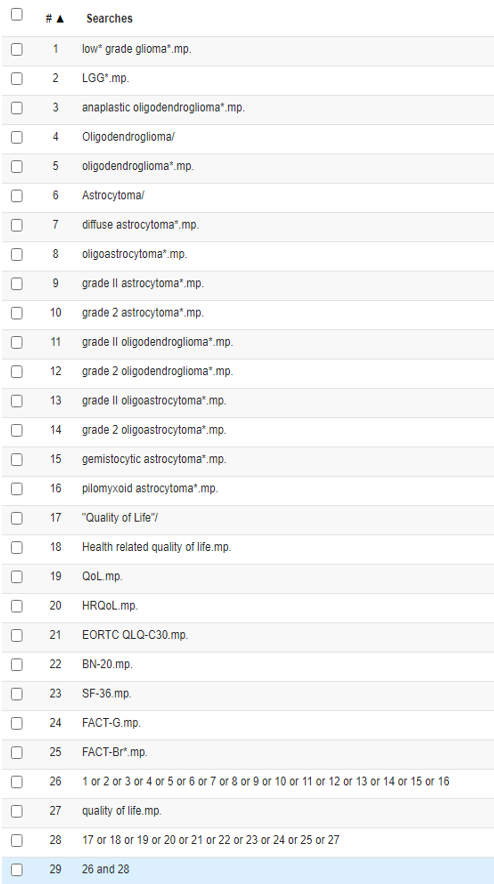


PsycINFO search


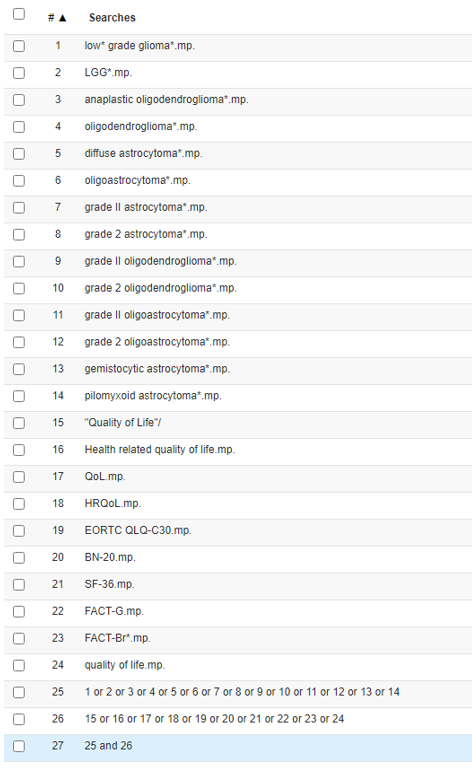


CINAHL search


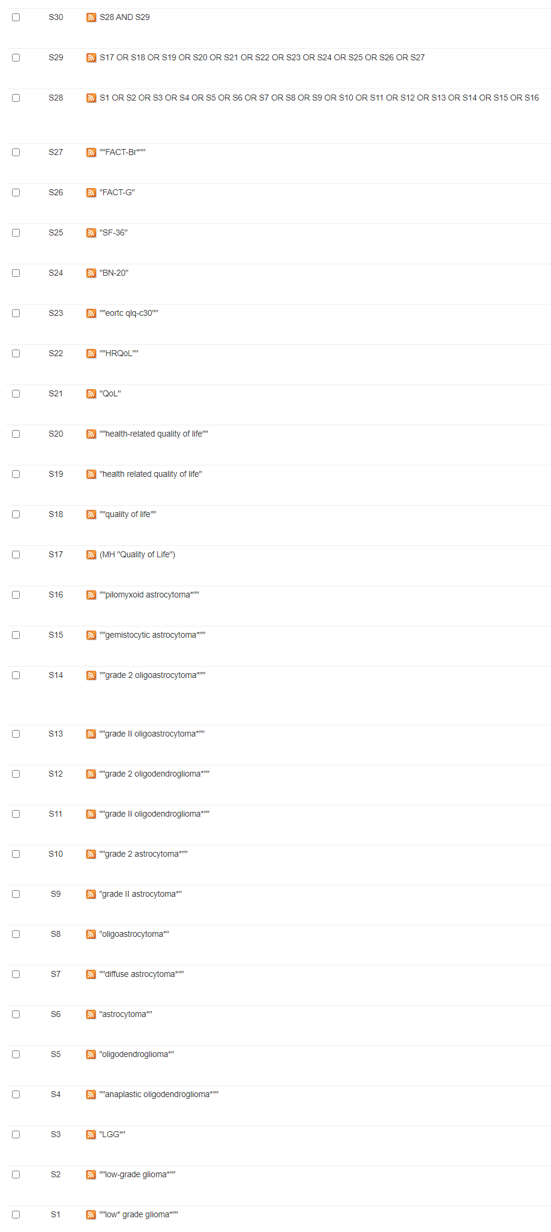


PubMed search


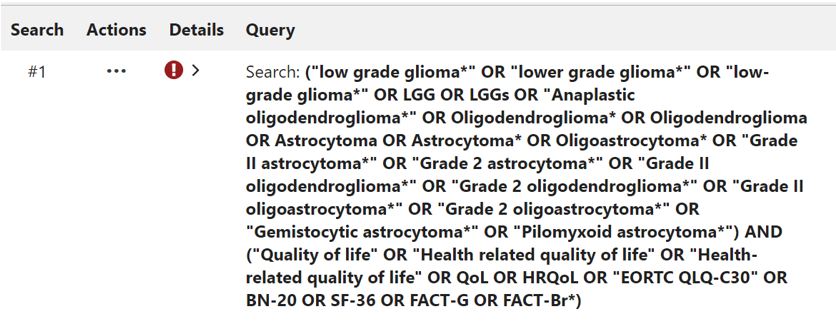


# Supplementary table 3: Additional population characteristics of included studies that assessed health-related quality of life in low-grade glioma patients

| **Study** | **Eligible population** | **Ethnicity** | **Socio-economic status^a^** | **Karnofsky performance status** | **Genetic markers** |
| --- | --- | --- | --- | --- | --- |
| Aaronson (2011); Boele (2014; 2015); Ediebah (2017) | *Inclusion:* no tumour recurrence for ≥1 year after diagnosis and primary treatment, no radiological signs of reccurence within 3 months prior to participation; *Exclusion:* corticosteroid users, non-Dutch speakers and people with severe neuropsychiatric deficits that were unable to communicate adequately. *Do not detail what determines severe neuropsychiatric deficits.* | NR | *Education:* mean level 4.2 (SD 2.1) | 88.1 (SD 13.6) | NR |
| Affronti (2018) | *Inclusion:* adults over 18; Pathological diagnosis of LGG (WHO grade II); enrolled at first 2 months after diagnosis; *Exclusion:* KPS <70 | White (93%), African American (7%) | NR | 70-80 (60%), 90-100 (40%) | IDH1 mutation (60%), IDH1 wild type (27%); TERT mutation (53%), TERT wild type (27%); 1p19q codeleted (40%), 1p19q intact (40%); MGMT methylated (63%), MGMT unmethylated (13%) |
| Budrukkar (2009) | *Inclusion:* every consecutive adult brain tumour patients (age ≥18 years) registered in the neuro-oncology clinic, histological subtypes of high-grade glioma, low-grade glioma and benign tumours; *Exclusion:* histopathology other than these three groups. | NR | NR^b^ | NR^b^ | NR |
| Campanella (2017) | *Inclusion:* patients showing radiologically stable LGG appearance at the time of evaluation, fluent in Italian, free from treatment for preceding psychiatric conditions;  *Exclusion:* LGGs who progressed toward a HGG. | NR | *Education:* mean 14.2 (8-17) years | NR | NR |
| Correa (2007; 2008) | *Inclusion:* not undergoing treatment or completed treatment >6 months prior to enrollment, no active disease (stable MRI); *Exclusion:* psychiatric or neurological disorders, not fluent in English. | NR | *Employment:* working (62.5%), not working (37.5%); *Education:* mean 16 years | NR | NR |
| Drewes (2018) | *Inclusion:* adults aged ≥18 years, undergoing first time surgery for glioma; *Exclusion:* further surgeries needed, missing forms, do not understand due to cognitive or language problems (*proxy ratings by close relatives used where possible if patient was not able to answer due to speech or cognitive impairment*). | NR | NR | ≥70 (95%), <70 (5%) | NR |
| Gabel (2019) | *Inclusion:* Histological diagnosis of a grade I-IV glioma, aged ≥18 years; *Exclusion:* Language and/or neurocognitive dysfunction limiting patient ability to participate. *Aphasia was assessed by the Boston Diagnostic Aphasia Examination. Cognitive function was screened using the Montreal Cognitive Assessment.* | NR | *Education:* completed college (71.4%), did not complete college (28.6%); *Employment:* employed at diagnosis (57.1%), unemployed (42.9%) | NR | NR |
| Gustafsson (2006) | *Inclusion:* Histologically verified LGG, aged ≥18 years, capable of completing the questionnaire (independent or with help); *Exclusion:* Severe cognitive problems resulting in obvious difficulty in communicating. *Do not detail what determines severe cognitive impairment.* | NR | NR | NR | NR |
| Jakola (2012) | *Inclusion:* Adult patients ≥18 years with histological verified supratentorial grade II glioma (diffuse astrocytomas, oligodendrogliomas and oligoastrocytomas); *Exclusion:* patients that did not receive a reliable grading | NR | NR | ≥80 (91%) | NR |
| Jiang (2019) | *Inclusion:* histological diagnosis of LGG (WHO grade II), age ≥18 years; *Exclusion:* severe cognitive/ neurological impairment preventing communication with the patient; a history of psychiatric disorders before the diagnosis of brain tumour; time elapse from the diagnosis of brain tumour to surgery >3 month. *Do not detail what determines severe cognitive impairment.* | NR | *Employment (before surgery):* working (62%), not working (38%); *Education:* <high school (32%), high school (53%), graduate degree (15%) | NR | IDH1 mutation: mutated (85%), wild type (15%); 1p/19q codeletion: detected (28%), Not detected (72%) |
| Kim (2020) | *Inclusion:* age ≥20 years, diagnosis of a primary glioma for more than 3 months, receiving follow-up care for 3 months since completion of treatments following diagnosis;  *Exclusion:* history of other major health issues that could influence quality of life or symptoms. | NR | NR^b^ | NR | NR |
| Klein (2003) | *Inclusion:* patients (1) with low-grade astrocytomas, oligodendrogliomas or oligoastrocytomas, (2) without clinical signs of tumour recurrence for at least 1 year after the histological diagnosis and primary treatment, and (3) without radiological signs of recurrence within 3 months before testing; *Exclusion:* if they used corticosteroids, did not have a basic proficiency in the Dutch language, were unable to communicate adequately, or received radiotherapy as secondary treatment. *Do not detail what determines an inability to communicate adequately.* | NR | *Education:* mean level 4.2 (2.2); 4.5 (2.2); 4.1 (1.8); 4 (2.3); 3.7 (2); 4.7 (2.1) | NR | NR |
| Leonetti (2021) | *Inclusion:* age ≥18 years, absence of severe comprehension deficits affecting the abilities to complete the questionnaires (tested using token test), absence of previous psychiatric symptoms or disease, absence of current medications for psychiatric conditions, histomolecular diagnosis of LGGs and HGGs, newly diagnosed glioma with no history of treatments;  *Exclusion:* tumour progression during the assessment period. | NR | *Education:* mean 13.9 (3.01) years | NR | IDH mutated (73.8%), IDH wildtype (26.3%) |
| Mahalakshmi (2015) | *Inclusion:* Patient diagnosed with a brain tumour, ≥18 years old, conscious and able to sign the consent forms; *Exclusion:* Suspected brain tumour patients before the confirmation, severe cognitive problems resulting in obvious difficulty in communicating, those who had other disease such as diabetes, neurodegeneration, and heart related problems. *Do not detail what determines severe cognitive problems.* | NR | NR^b^ | NR^b^ | NR |
| Okita (2015) | *Inclusion:* grade II glioma with no evidence of active disease, or receipt of radiotherapy and chemotherapy at the time of participation. | NR | NR | 100 (16%),  90 (58%),  80 (16%),  70 (10%) | NR |
| Reijneveld (2001) | *Inclusion:* all patients were at least 18 years old, presented with epileptic seizures without neurologic deficits, showed non-enhancing supratenorial lesions without edema, no radiologic or clinical signs of progression for >6 months from presumed diagnosis. | NR | *Education:* mean level 3.5 | 88.7 (SD 9.7) | NR |
| Ruge (2011) | *Inclusion:* newly diagnosed, untreated supratentorial LGG; *Exclusion:* diagnosis other than WHO grade II astrocytoma, oligodendroglioma, and oligoastrocytoma. | NR | *Employment:* employed at time of diagnosis (91%) | 83.6 (SD 6.5) | NR |
| Salo (2002); Mainio (2006) | *Inclusion:* adult >16 years old with a primary brain tumour diagnosis; *Exclusion:* those with metastases | NR | NR | NR | NR |
| Teng (2021) | *Inclusion:* age >18 years, undergone biopsy or resection of an intracranial LGG and were in routine follow-up;  *Exclusion:* patients with other brain or spine lesions, previous malignancy, Grade III or IV gliomas, or neurofibromatosis type 1 or 2; need to complete questionnaires independently in English. | NR | NR | NR | NR |
| Umezaki (2020) | *Inclusion:* adult patients with WHO grade II–IV glioma according to 2016 classification; *Exclusion:* those who could not answer the questionnaire because of impaired consciousness or aphasia. *Do not detail how aphasia was determined.* | NR | NR^b^ | NR^b^ | IDH-mutant (n=13), IDH-wildtype (n=3), 1p19-codeleted (n=9), not otherwise specified (n=15) |
| Wang (2018); Li (2019a; 2019b) | *Inclusion:* histologically confirmed diagnoses of WHO grade I or II glioma, ≥18 years old; KPS >60; *Exclusion:* abnormal cognition MMSE ≤24, unable to read or understand the questionnaire. *Excludes those with at least mild cognitive impairment, determined by MMSE, despite impact to cognitive function being common in this population.* | NR | *Insurance:* yes (62.7%), no (37.3%) | >60 (100%) | NR |
| Yavas (2012) | *Inclusion:* >18 years of age and had a histological diagnosis of a primary low-grade brain tumour; *Exclusion:* patients who were not able to read or write the questionnaires, who had severe cognitive impairment resulting in obvious difficulty in communicating, whose KPS were <70 and/or whose expected survival time <3 months. *Do not detail what determines significant cognitive impairment; excluding these participants despite cognitive function being a primary measure.* | NR | *Education (literary status):* primary (20.93%), middle (18.6%), high (13.95%), university (46.51%) | 100 (39.53%),  90 (48.84%),  80 (6.98%) | NR |
| ^a^Socio-economic status was assessed based on education, employment, monthly income, and insurance status.  ^b^This demographic was not reported separately for low-grade gliomas. | | | | | |

# Supplementary table 4: Health-related quality-of-life instruments and their scoring

| **Instrument** | **Citation** | **Dimensions assessed** | **Scoring** |
| --- | --- | --- | --- |
| BN20 | Osoba D, Aaronson NK, Muller M, et al. The development and psychometric validation of a brain cancer quality-of-life questionnaire for use in combination with general cancer-specific questionnaires. *Qual Life Res 1996 51*. 1996;5(1):139-150. | Future uncertainty, visual disorder, motor function, communication deficit, headache, seizures, drowsiness, hair loss, itchy skin, leg weakness, bladder control | Subscale scores. Higher score indicates worse QoL. Four-point scale from 1 (Not at all) to 4 (Very much) for how much experienced an issue in the past week. |
| EQ-5D | Rabin R, Charro F de. EQ-SD: a measure of health status from the EuroQol Group. *Ann Med*. 2001;33(5):337-343. | Mobility, self-care, usual activities, pain/ discomfort, anxiety/ depression | Individual score. Higher score indicates better QoL. Visual analogue scale from 0 (Worst imaginable health state) to 100 (Best imaginable health state). |
| FACT-Br | Weitzner MA, Meyers CA, Gelke CK, Byrne KS, Levin VA, Cella DF. The Functional Assessment of Cancer Therapy (FACT) scale. Development of a brain subscale and revalidation of the general version (FACT‐G) in patients with primary brain tumors. *Cancer*. 1995;75(5):1151-1161. | Physical well-being, social/family well-being, emotional well-being, functional well-being, brain cancer subscale | Subscale and total scores. Higher score indicates better QoL. Five-point scale from 0 (Not at all) to 4 (Very much so) for truth of a statement in the past week. |
| FACT-Cog | Wagner LI, Lai JS, Cella D, Sweet J, Forrestal S. Chemotherapy-related cognitive deficits: development of the FACT-Cog instrument. *Ann Behav Med*. 2004;27(Suppl 10). | Perceived cognitive impairments, impact of perceived cognitive impairments on QoL, comments from others, perceived cognitive abilities | Subscale and total scores. Higher score indicates better QoL. Five-point scale from 0 (Never) to 4 (Several times a day) for how much experienced an issue in the past week. |
| FACT-G | Cella DF, Tulsky DS, Gray G, et al. The Functional Assessment of Cancer Therapy scale: development and validation of the general measure. *J Clin Oncol*. 1993;11(3):570-579. | Physical well-being, social/family well-being, emotional well-being, functional well-being | Subscale and total scores. Higher score indicates better QoL. Five-point scale from 0 (Not at all) to 4 (Very much) for how much experienced an issue in the past week. |
| FACIT-Fatigue | Yellen SB, Cella DF, Webster K, Blendowski C, Kaplan E. Measuring fatigue and other anemia-related symptoms with the Functional Assessment of Cancer Therapy (FACT) measurement system. *J Pain Symptom Manage*. 1997;13(2):63-74. | Physical well-being, social/family well-being, emotional well-being, functional well-being, fatigue | Subscale and total scores. Higher score indicates better QoL. Five-point scale from 0 (Not at all) to 4 (Very much) for how much experienced an issue in the past week. |
| Neuro-QoL | Gershon RC, Lai JS, Bode R, et al. Neuro-QOL: quality of life item banks for adults with neurological disorders: item development and calibrations based upon clinical and general population testing. *Qual Life Res*. 2012;21(3):475-486. | Anxiety, depression, fatigue, motor function, cognitive function, emotional/ behavioural dyscontrol, sleep disturbance, positive affect and well-being, social role, stigma | Subscale scores. Higher score indicates worse QoL (emotional and behavioural dyscontrol) and better QoL (cognitive function). Five-point scale from 0 (Not at all) to 4 (Very much) for how much experienced an issue in the past week. |
| NIH-PROMIS | Broderick JE, DeWitt EM, Rothrock N, Crane PK, Forrest CB. Advances in patient-reported outcomes: the NIH PROMIS® measures. *Egems*. 2013;1(1). | Pain, fatigue, physical functioning, emotional distress, social role participation | Subscale scores. Unclear whether higher or lower score indicates better QoL. Five-point scale from 1 to 5 (anchors vary). |
| Nottingham Health Profile | Hunt SM, McKenna SP, McEwen J, Williams J, Papp E. The Nottingham Health Profile: subjective health status and medical consultations. *Soc Sci Med Part A Med Psychol Med Sociol*. 1981;15(3):221-229. | Pain, mobility, social isolation, energy, emotion, sleep | Subscale scores. Higher score indicates worse QoL. Weighted ‘yes’ or ‘no’ questions for a score 0-100 for whether something is a problem. |
| PWB | Ryff CD, Keyes CLM. The structure of psychological well-being revisited. *J Pers Soc Psychol*. 1995;69(4):719. | Psychological wellbeing | Subscale and total scores. Higher scores indicate better psychological wellbeing. Six-point scale from 1 (strongly disagree) to 6 (strongly agree) |
| QLQ-C30 | Aaronson NK, Ahmedzai S, Bergman B, et al. The European Organization for Research and Treatment of Cancer QLQ-C30: a quality-of-life instrument for use in international clinical trials in oncology. *JNCI J Natl Cancer Inst*. 1993;85(5):365-376. | Physical, role, emotional, cognitive, social functioning, fatigue, nausea, pain, dyspnoea, insomnia, appetite loss, constipation, diarrhoea, financial difficulties | Subscale and total scores. Higher functioning score indicates better QoL. Higher symptom score indicates worse QoL. Four-point scale from 1 (Not at all) to 4 (Very much) for how much experienced an issue in the last week. |
| SF-36 | Aaronson NK, Muller M, Cohen PDA, et al. Translation, validation, and norming of the Dutch language version of the SF-36 Health Survey in community and chronic disease populations. *J Clin Epidemiol*. 1998;51(11):1055-1068. | Physical functioning, role functioning - physical, bodily pain, general health, vitality, social functioning, role functioning - emotional, mental health | Subscale scores. Higher score indicates better QoL. Variety of scales and endpoints used. |
| Sin Tonen’s 15D | Sintonen H. The 15D instrument of health-related quality of life: properties and applications. *Ann Med*. 2001;33(5):328-336. | Mobility, vision, hearing, breathing, sleeping, eating, speech, excretion, usual activities, mental function, discomfort and symptoms, depression, distress, vitality, sexual activity | Total score. Higher score indicates better QoL. Index score ranges from 0 (being dead) to 1 (no problems). Five-point scales (anchors vary) for describing present health status. |
| **Abbreviations:** *BN-20 = European Organisation for Research and Treatment of Cancer Quality of life Questionnaire Brain Neoplasm; EQ-5D = EuroQoL 5 dimension; FACT-Br = Functional Assessment of Cancer Therapy - Brain; FACT-Cog = Functional Assessment of Cancer Therapy – Cognitive function; FACT-G = Functional Assessment of Cancer Therapy – General; FACIT-Fatigue = Functional Assessment of Chronic Illness Therapy - Fatigue; NIH-PROMIS = National Institutes of Health - Patient Reported Outcomes Measurement Information System; Neuro-QoL = Quality of Life in Neurological Disorders; PWB = Psychological wellbeing scale; QLQ-C30 = European Organisation for Research and Treatment of Cancer Quality of life Questionnaire Core; SF-36 = Short Form 36 health survey questionnaire* | | | |

# Supplementary table 5: Quality appraisal

| **Appraisal items** | Aaronson (2011)^a^ | Boele (2014)^a^ | Boele (2015)^a^ | Ediebah (2017)^a^ | Affronti (2018) | Budrukkar (2009) | Campanella (2017) | Correa (2007)^b^ | Correa (2008)^b^ | Drewes (2018) | Gabel (2019) | Gustafsson (2006) | Jakola (2012) | Jiang (2019) | Kim (2020) |
| --- | --- | --- | --- | --- | --- | --- | --- | --- | --- | --- | --- | --- | --- | --- | --- |
| 1. Clearly stated aims | 2 | 1 | 2 | 2 | 2 | 2 | 2 | 2 | 2 | 2 | 1 | 2 | 2 | 2 | 2 |
| 2. Participant eligibility and recruitment strategy clearly documented | 1 | 1 | 1 | 1 | 2 | 2 | 2 | 2 | 2 | 2 | 2 | 1 | 2 | 1 | 2 |
| 3. Main features of population/design described | 2 | 2 | 2 | 2 | 2 | 1 | 1 | 2 | 2 | 2 | 1 | 2 | 2 | 2 | 0 |
| 4. Non-responders (and non-participants) described | 2 | 2 | 2 | 2 | 1 | 1 | 1 | 2 | 2 | 2 | 0 | 2 | 1 | 1 | 1 |
| 5. Presence of a control group | 2 | 2 | 2 | 0 | 0 | 1 | 1 | 0 | 0 | 1 | 1 | 0 | 0 | 0 | 1 |
| 6. Main limitations identified and acceptable | 2 | 1 | 2 | 2 | 1 | 1 | 2 | 1 | 1 | 2 | 2 | 2 | 2 | 1 | 2 |
| 7. Sample size justified | 0 | 0 | 0 | 0 | 0 | 0 | 0 | 0 | 0 | 0 | 0 | 0 | 0 | 0 | 0 |
| 8. No evidence of selective reporting of results | 2 | 2 | 2 | 2 | 2 | 2 | 2 | 1 | 1 | 2 | 2 | 2 | 1 | 2 | 2 |
| 9. Statistical methods described | 2 | 2 | 2 | 2 | 1 | 1 | 2 | 2 | 2 | 2 | 2 | 1 | 2 | 2 | 2 |
| 10. Statistical methods appropriate | 2 | 2 | 2 | 2 | 1 | 1 | 2 | 2 | 2 | 2 | 2 | 1 | 2 | 2 | 2 |
| 11. Measures relevant, validated and described adequately | 2 | 2 | 2 | 2 | 1 | 2 | 2 | 1 | 1 | 2 | 2 | 2 | 2 | 2 | 2 |
| 12. Results discussed adequately | 2 | 1 | 2 | 2 | 2 | 2 | 1 | 0 | 0 | 2 | 2 | 2 | 1 | 2 | 1 |
| **Total score** | **21** | **18** | **20** | **19** | **15** | **16** | **18** | **15** | **15** | **21** | **17** | **17** | **17** | **17** | **17** |

Supplementary table 5 continued.

| **Appraisal items** | Klein (2003) | Leonetti (2021) | Mahalakshmi (2015) | Okita (2015) | Reijneveld (2001) | Ruge (2011) | Salo (2002)^c^ | Mainio (2006)^c^ | Teng (2021) | Umezaki (2020) | Wang (2018)^d^ | Li (2019a)^d^ | Li (2019b)^d^ | Yavas (2012) | |
| --- | --- | --- | --- | --- | --- | --- | --- | --- | --- | --- | --- | --- | --- | --- | --- |
| 1. Clearly stated aims | 2 | 2 | 2 | 2 | 2 | 2 | 2 | 2 | 2 | 2 | 2 | 2 | 2 | 2 | |
| 2. Participant eligibility and recruitment strategy clearly documented | 1 | 2 | 1 | 1 | 2 | 2 | 2 | 2 | 2 | 1 | 2 | 2 | 2 | 1 | |
| 3. Main features of population/design described | 2 | 2 | 1 | 2 | 1 | 2 | 1 | 1 | 1 | 1 | 2 | 2 | 2 | 2 | |
| 4. Non-responders (and non-participants) described | 2 | 1 | 2 | 1 | 1 | 2 | 2 | 0 | 0 | 2 | 2 | 2 | 2 | 0 | |
| 5. Presence of a control group | 1 | 1 | 1 | 0 | 1 | 1 | 1 | 0 | 1 | 1 | 0 | 0 | 0 | 0 | |
| 6. Main limitations identified and acceptable | 0 | 2 | 2 | 1 | 1 | 0 | 0 | 1 | 2 | 2 | 2 | 2 | 2 | 1 | |
| 7. Sample size justified | 0 | 0 | 0 | 0 | 0 | 0 | 0 | 0 | 0 | 0 | 2 | 0 | 0 | 0 | |
| 8. No evidence of selective reporting of results | 2 | 1 | 2 | 2 | 0 | 2 | 2 | 2 | 1 | 2 | 2 | 2 | 2 | 2 | |
| 9. Statistical methods described | 2 | 2 | 2 | 2 | 2 | 2 | 2 | 2 | 2 | 2 | 2 | 2 | 2 | 2 | |
| 10. Statistical methods appropriate | 2 | 2 | 2 | 2 | 2 | 2 | 2 | 2 | 2 | 2 | 2 | 2 | 2 | 2 | |
| 11. Measures relevant, validated and described adequately | 2 | 2 | 2 | 2 | 2 | 2 | 2 | 2 | 2 | 2 | 2 | 2 | 2 | 2 | |
| 12. Results discussed adequately | 2 | 1 | 2 | 2 | 1 | 2 | 0 | 1 | 1 | 2 | 1 | 2 | 2 | 1 | |
| **Total score** | **18** | **18** | **19** | **17** | **15** | **19** | **16** | **15** | **16** | **19** | **21** | **20** | **20** | **15** | |
| **Score Key:** *Yes* = Score of 2; *Partial* = Score of 1; *No* = Score of 0  **Scoring:** *Good quality* = 17-24; *Acceptable quality* = 9-16; *Low quality* = 0-8  ^a^Aaronson (2011), Boele (2014; 2015), and Ediebah (2017) report from the same study.  ^b^Correa (2007; 2008) report from the same study.  ^c^Salo (2002) and Mainio (2006) report from the same study.  ^d^Wang (2018) and Li (2019a; 2019b) report from the same study. | | | | | | | | | | | | | | |  |

# Supplementary data: Overall health-related quality-of-life findings

See ‘Supplementary data_HRQoL findings’ spreadsheet.

The ‘HRQoL’ tab shows findings listed by study in alphabetical order.

The ‘HRQoL_grouped’ tab shows findings grouped by HRQoL instrument used.
